# Long-Term Follow-Up of Gemogenovatucel-T (Vigil) Survival and Molecular Signals of Immune Response in Recurrent Ovarian Cancer

**DOI:** 10.3390/vaccines9080894

**Published:** 2021-08-12

**Authors:** Rodney P. Rocconi, Laura Stanbery, Luciana Madeira da Silva, Robert A. Barrington, Phylicia Aaron, Luisa Manning, Staci Horvath, Gladice Wallraven, Ernest Bognar, Adam Walter, John Nemunaitis

**Affiliations:** 1 Mitchell Cancer Institute, Division of Gynecologic Oncology, University of South Alabama, Mobile, AL 36604, USA; rocconi@health.southalabama.edu; 2 Gradalis, Inc., 2545 Golden Bear Drive, Suite 110, Carrollton, TX 75006, USA; lnejedlik@gradalisinc.com (L.S.); paaron@gradalisinc.com (P.A.); lmanning@gradalisinc.com (L.M.); shorvath@gradalisinc.com (S.H.); gwallraven@gradalisinc.com (G.W.); ebognar@gradalisinc.com (E.B.); 3 Department of Microbiology and Immunology, University of South Alabama, Mobile, AL 36688, USA; lsilva@health.southalabama.edu (L.M.d.S.); rbarrington@health.southalabama.edu (R.A.B.); 4 Promedica, Sylvania, OH 43560, USA; adam.walterMD@promedica.org

**Keywords:** Vigil, immune response, NanoString, TIS, gene expression profile, immunotherapy, ovarian cancer

## Abstract

Aim: To determine the relationship between gene expression profile (GEP) and overall survival (OS) by NanoString following treatment with Vigil. Patients and Methods: Recurrent ovarian cancer patients (*n* = 21) enrolled in prior clinical trials. Results: GEP stratified by TIS^HIGH^ vs. TIS^LOW^ demonstrated OS benefit (NR vs. 5.8 months HR 0.23; *p* = 0.0379), and in particular, MHC-II elevated baseline expression was correlated with OS advantage (*p* = 0.038). Moreover, 1-year OS was 75% in TIS^HIGH^ patients vs. 25% in TIS^LOW^ (*p* = 0.03795). OS was also correlated with positive γ-IFN ELISPOT response, 36.8 vs. 23.0 months (HR 0.19, *p* = 0.0098). Conclusion: Vigil demonstrates OS benefit in correlation with TIS^HIGH^ score, elevated MHC-II expression and positive γ-IFN ELISPOT in recurrent ovarian cancer patients.

## 1. Background

Ovarian cancer remains a complex and difficult condition to treat, in part because of the advanced stage at presentation. Using American Cancer Society estimates, 21,750 new cases of ovarian cancer are expected and 13,940 deaths from disease are estimated in the USA in 2020 [1]. With optimal standard of care treatment, including surgical debulking and adjuvant or neoadjuvant chemotherapy consisting of paclitaxel and carboplatin with or without bevacizumab in newly diagnosed patients with advanced surgically resectable disease, 5-year survival rates are only 48% [2,3]. Patients with stage IV disease have even worse survival, with 5-year rates below 20% [4]. Additionally, the majority of advanced-stage ovarian cancer patients relapse within 2 years [2]. Research has involved developing improved maintenance regimens, which provide improvements in progression-free survival (PFS) [5,6]. In particular, poly (ADP-ribose) polymerase (PARP) inhibitors have shown benefit in prolonging PFS; however, this benefit is predominately in the *BRCA1/2* mutant population, with limited efficacy in *BRCA1/2* wild-type individuals [7,8,9,10]. Prognosis in recurrent disease patients unfortunately is much worse; median survival is near 2 years and focus of management is on quality of life support. Recurrent disease patients are rarely curable, although a recent comparison of platinum-sensitive recurrent ovarian cancer patients with the *BRCA1/2* mutation in the SOLO-2 study revealed a 5-year overall survival (OS) of 41.6% with the use of olaparib as second-line or greater maintenance compared to standard of care of 33.3% [11].

Vigil is constructed using harvested autologous tumor tissue and given as an intradermal injection in order to access personal neoantigen display. Tumor cells are transfected with a plasmid containing the GM-CSF gene and a bifunctional short hairpin RNA which targets furin [12]. Successful furin knockdown is demonstrated by downstream inhibition of TGFβ1 and TGFβ2, potent immune suppressor cytokines, which have been shown to improve the anticancer immune response when suppressed [12]. Further, immune function and enhanced antigen expression is provided with exogenous GM-CSF production [13]. Vigil has also been shown to increase CD3+/CD8+ circulating mononuclear cells in solid tumor patients [14]. Personal neoantigen display and T cell priming and expansion may point to memory T cell generation by Vigil.

Previous results have been reported from a Phase I trial in late-stage cancer patients, involving 19 different solid tumor types, who received two different dose levels of Vigil (1 × 10^7^ and 2.5 × 10^7^ cells/injection) [15]. Safety confirmed at both dose levels demonstrated no dose-dependent toxic responses. Previous long-term follow-up of nearly three years identified that γ-IFN-ELISPOT positivity was correlated with OS advantage to Vigil treatment [16]. Moreover, results from Phase IIa testing of Vigil vs. placebo [17,18] and recently from a Phase IIb clinical trial involving newly diagnosed advanced ovarian cancer patients with a *BRCA1/2* wild-type genetic profile showed greater clinical benefit, as both relapse-free survival (RFS) and OS were improved [19].

Limited effectiveness of immunotherapy, however, has been seen in ovarian cancer [20,21,22]. Nonetheless, subsets of patients exhibit durable responses that can exceed 2 years. Several biomarkers have been studied, but no demonstration of distinguishing signals between responders and non-responders has been shown [10,11,12,23,24]. RNA-based evaluations suggest that specific mutational load, cytolytic activity and neoantigen signatures offer potential predictive indication with immunotherapy [25]. Here, we focus on recurrent ovarian cancer patients and provide additional long-term follow-up including a molecular biomarker profile of this cohort of patients.

## 2. Materials and Methods

### 2.1. Study Design

Vigil plasmid construction and cGMP manufacturing have been previously described [12,15,17]. Tumor tissue was excised and processed according to protocol guidelines and shipped to Gradalis, Inc. for vaccine manufacturing. Tissue was processed and transfected as previously described [15]. All recurrent ovarian cancer patients received Vigil at 1 × 10^7^ or 2.5 × 10^7^ cells/injection and were monitored closely for safety during study treatment as described [15]. Long-term follow-up was performed by phone survey and by medial record review. Trials were previously registered as NCT01061840 and NCT01309230.

### 2.2. ELISPOT Assay

ELISPOT was performed as previously described [15,17]. The Enzyme-Linked Immunospot Assay for Interferon Gamma (BD Biosciences, San Jose, CA, USA) was used. A sample was considered positive if >10 spots or 2× baseline was observed. ZellNet Consulting, Fort Lee, NJ provided quantitation.

### 2.3. RNA Isolation and Gene Expression Analysis

Pretreatment clinical specimens were collected as specified in the clinical protocol and consisted of frozen cells retained from fresh tissue that were harvested at time of tissue procurement. Total RNA was isolated using RNeasy Mini Kit (Qiagen, Venlo, The Netherlands). Gene expression analysis was conducted using the NanoString^®^ PanCancer Immuno-Oncology 360^TM^ CodeSet using the nCounter^®^ SPRINT platform (NanoString^®^ Technologies, Seattle, WA, USA). This unique 770-plex gene expression panel, which profiles the immune system, tumor and tumor microenvironment, was utilized to characterize individual genes and pathways that shape tumor–immune interactions. An incorporated algorithm of 18 specific functional genes known to be associated with immunotherapy response was used to calculate a tumor inflammation score (TIS) by the Nanostring^®^ IO360 Data Analysis Service, in addition to 42 signatures measuring important tumor immune activities and immune cell populations. The weighted scores used for calculation of the TIS and other signatures are NanoString^®^ intellectual property. Differential gene expression analysis between TIS^high^ (>6.0) and TIS^low^ (<6.0) samples was performed using the nSolver^TM^ Analysis Software v4.0 and the nSolver Advanced Analysis package with Benjamin–Yakhteh adjusted *p*-values. Pathway scores obtained in the nSolver Advanced Analysis were analyzed by Graphpad Prism. Good responders were defined as OS >12 months and poor responders ≤12 months. T-tests with Welch’s correction were used for comparison between groups. Values of *p* < 0.05 were considered significant. Heatmaps of signature scores were built using ClustVis (https://biit.cs.ut.ee/clustvis/, accessed on 3 August 2021) [26].

### 2.4. Statistics

Survival was analyzed using Graphpad Prism version 8.3.0 (GraphPad Software, Inc., San Diego, CA, USA) software to generate Kaplan–Meier curves and compare ELISPOT results, which included all recurrent/refractory patients enrolled. OS of patients still alive was censored using the last known date alive and was calculated from time of surgery/tissue procurement. The hazard ratios (HR) of OS analysis were estimated via a log-rank hazards ratio model. ELISPOT analysis compared ELISPOT+ and ELISPOT- results using a log-rank hazards ratio model. A one-sided *p*-value of 0.05 or less (log-rank) was considered to indicate statistical significance.

## 3. Results

### 3.1. Patient Demographics

Patient demographics are listed in Table 1. Twenty-one patients were enrolled in the Vigil studies [12,15,16] from May 2010 to December 2014. Patients had received a mean of 2.95 lines of prior systemic therapy as standard of care (range 1–10). A total of 124 vaccine doses were administered. The mean number of Vigil doses administered was 5.9 (range of 1–12). There was no difference in patient demographics including age between the overall population and those undergoing NanoString^®^. At recurrence, prior to study enrollment, patients received a variety of standard of care chemotherapy regimens including carboplatin, cisplatin, gemcitabine and paclitaxel among others. No patients received PARP inhibitors.

### 3.2. Overall Long-Term Survival and Safety

**Figure 1:**
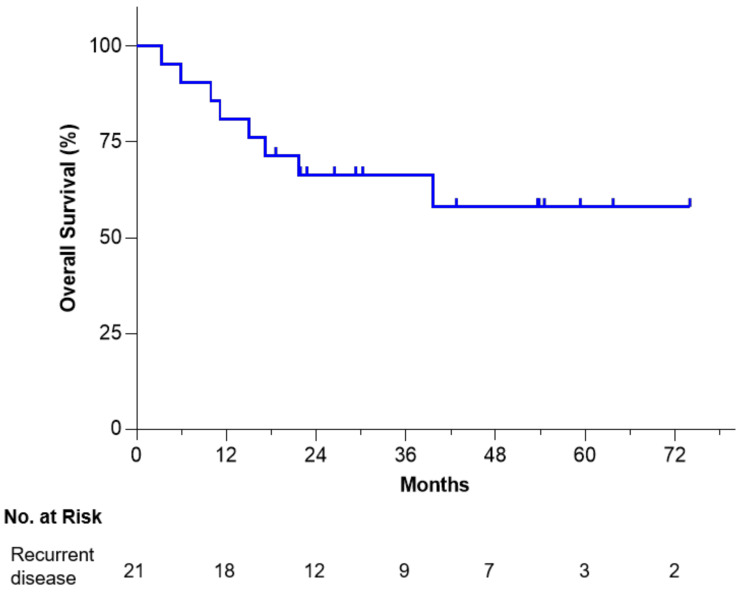
OS of Vigil-treated recurrent/refractory ovarian cancer patients from time of procurement.

As shown in Figure 1, Kaplan–Meier analysis of 21 recurrent ovarian cancer patients revealed a 58% survival rate at 6 years from time of surgery/tissue procurement, which encouragingly demonstrates a plateau maintained for just over 3 years. No long-term serious adverse events or Grade 3/4 Vigil-related toxic effects were observed or reported.

### 3.3. Immune Response Correlation to Overall Survival

**Figure 2:**
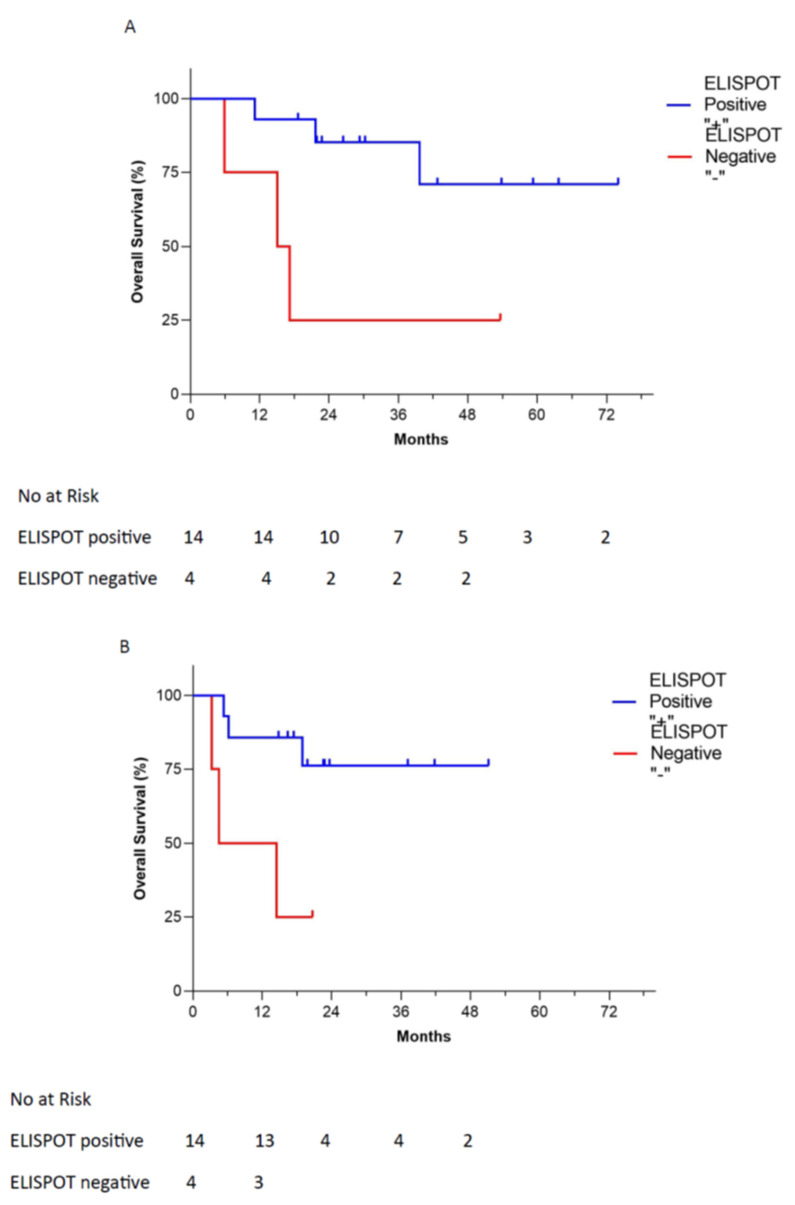
Overall survival relationship of Vigil treatment recurrent/refractory ovarian cancer patients by γ-IFN-ELISPOT-positive vs. γ-IFN-ELISPOT-negative recurrent ovarian cancer patients from time of tissue procurement (**A**) and start of treatment (**B**).

Twenty-one patients were assayed for γ-IFN-ELISPOT response during Vigil administration: 14 patients were shown to be γ-IFN-ELISPOT positive, four patients were negative and three were not able to be assessed. Of the four γ-IFN-ELISPOT-negative patients, two died from disease during the study: one cause of death was unknown and one patient was still alive. From time of tissue procurement (Figure 2A), median OS in γ-IFN-ELISPOT positive patients was not reached versus 16.1 months for ELISPOT negative patients (*p* = 0.0098, HR 0.19, estimated 95% CI: 0.021–1.7). OS from time of treatment start was similarly improved in γ-IFN-ELISPOT-positive patients; median was not reached versus 9.5 months, respectively (*p* = 0.0079, HR 0.18, estimated 95% CI: 0.019–1.7) (Figure 2B).

### 3.4. Immune Gene Expression Profiling

We explored TIS profiles and other gene expression signatures in 12 (who had sufficient tissue available) of the 21 recurrent ovarian cancer patients to assess the ability to detect immune-responsive (“hot”) tumors and how it correlates with clinical outcomes in response to Vigil. To apply the TIS score as a tool for patient enrichment, the pre-specified consensus threshold of 6.0 was used [27].

**Figure 3:**
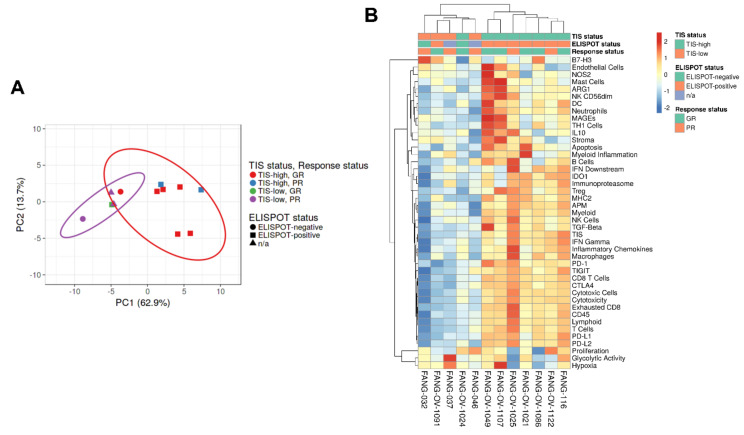
Principal component analysis (PCA) was completed to detect intrinsic clusters between responders to Vigil treatment and γ-IFN-ELISPOT reactivity post-Vigil treatment as well as possible outliers. TIS^HIGH^ good responders (Tumor inflammation score (TIS)) > 6, OS > 12months) post-Vigil treatment = red; TIS^HIGH^ poor responders (TIS score > 6, OS < 12months) post-Vigil treatment = blue; TIS^LOW^ good responders (TIS score < 6, OS > 12months) post-Vigil treatment = green; TIS^LOW^ poor responders (TIS score < 6, OS < 12months) post-Vigil treatment = purple; γ-IFN-ELISPOT-negative = circles; γ-IFN-ELISPOT-positive = squares; post-Vigil; γ-IFN-ELISPOT not evaluable = triangle (**A**) Heatmap of immune pathways stratified by TIS status, response to Vigil therapy and γ-IFN-ELISPOT status. Blue scale indicates under-expressed genes and red scale upregulated genes (**B**).

Principal component analysis (PCA) was performed to identify the distribution of signature scores of TIS^HIGH^ (red) and TIS^LOW^ tumors (blue), good responders (red) and poor responders (blue), γ-IFN-ELISPOT-negative (circles) and γ-IFN-ELISPOT-positive (squares) and γ-IFN-ELISPOT not evaluable (triangles) (Figure 3A). This analysis shows that the distance between each dot is related to the similarity between observations in high-dimensional space. From these data, we assume that the signature scores associated with patient response to Vigil (good response (GR) vs. poor response (PR)) and γ-IFN-ELISPOT reactivity (positive/negative) are strongly conserved. The 43 signature scores for each patient are presented on a heatmap in Figure 3B. The scores are grouped by TIS grouping, response status to Vigil and γ-IFN-ELISPOT reactivity after Vigil treatment. The heatmap shows TIS grouping of >/<6.0, and patients who demonstrated a positive or negative γ-IFN-ELISPOT response are clearly separated. The majority of TIS^HIGH^ tumors were associated with γ-IFN-ELISPOT positivity (FANG-OV-1024 was γ-IFN-ELISPOT-negative). Most good responders were associated with γ-IFN-ELISPOT positivity, with the exception of FANG-OV-1091 (γ-IFN-ELISPOT-negative).

**Figure 4:**
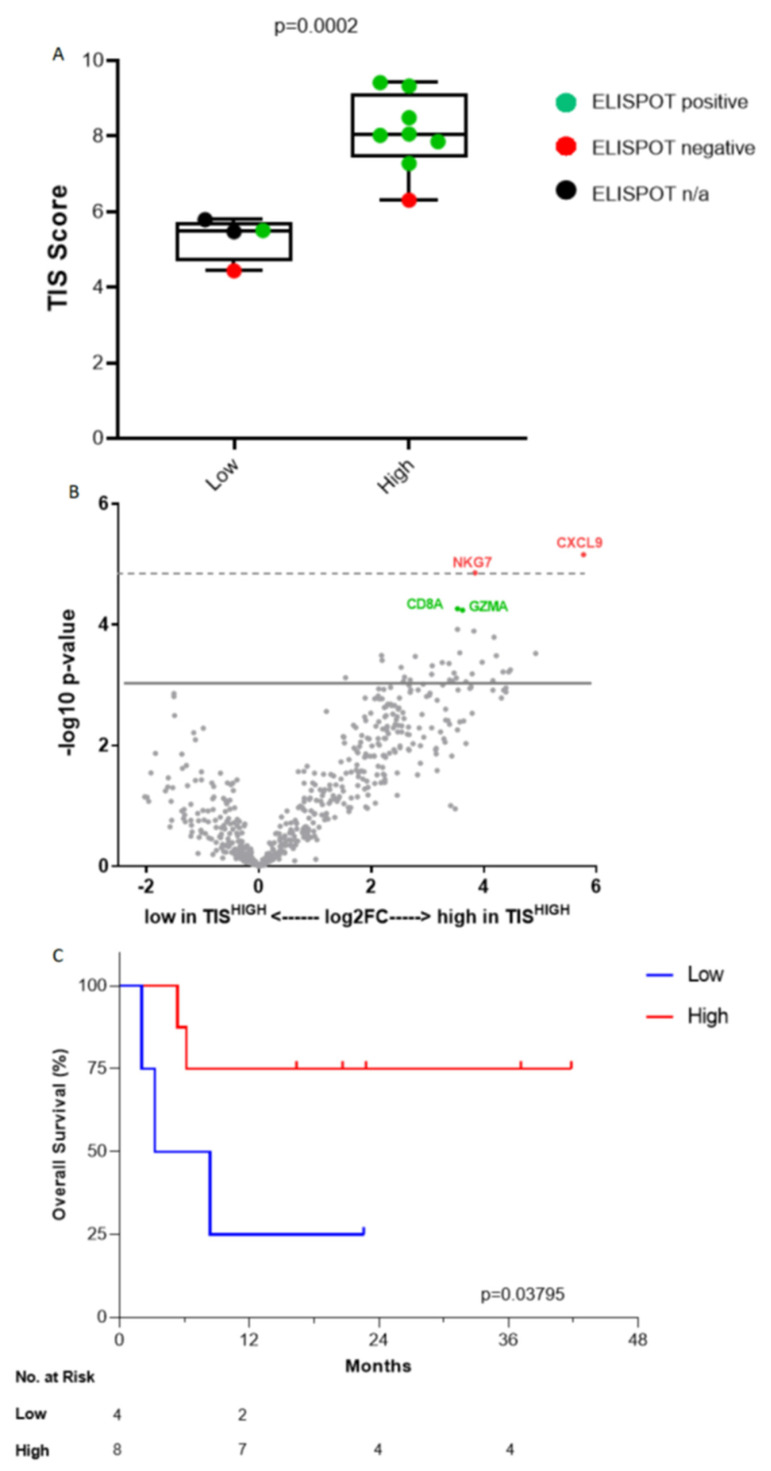
γ-IFN-ELISPOT reactivity stratified by tumor inflammation score^HIGH^ (TIS) vs. TIS^LOW^. Whiskers represent minimum and maximum TIS scores. Statistical analyses of TIS scores were performed using unpaired T-tests with Welch correction. (**A**) Volcano plot of *p*-value versus log2 fold change in the differential expression between TIS^HIGH^ and TIS^LOW^. The test for differential expression was done by fitting the log2 normalized count to the response with linear model. The *p*-values were adjusted by the Benjamini and Yekutieli (BY) adjustment. Dots corresponding to genes that are significant at *p* < 0.5 (dashed line) are labeled in red. Solid line represents *p* < 0.10 (**B**). Overall survival relationship of Vigil treatment stratified by TIS^HIGH^ vs. TIS^LOW^ (**C**).

Immune gene signatures of patient tumors demonstrated the ability of TIS to detect “hot” tumors, with a significant correlation of TIS^HIGH^ tumors to ELISPOT-positive y-IFN-producing samples (as shown in Figure 4A, *p* = 0.0002). Significant immune GEP differences stratified by TIS^HIGH^ vs. TIS^LOW^ included MHC-II (*p* = 0.017), γ-IFN (*p* = 0.001), TGFβ (*p* = 0.011), IDO1 (*p* = 0.023), PD-1 (*p* = 0.002), PD-L1 (*p* = 0.004) and PD-L2 (*p* = 0.0001) signatures. Significant cellular GEP differences stratified by TIS^HIGH^ vs. TIS^LOW^ included CD8 T cells (*p* = 0.0001), cytotoxic cells (*p* < 0.0001), lymphoid cells (*p* = 0.001), dendritic cells (*p* = 0.003), macrophages (*p*=0.003), neutrophils (*p* = 0.004), myeloid cells (*p* = 0.002), NK cells (*p* = 0.007) and T cells (*p* = 0.003) A heatmap of twenty-seven significant signature scores is provided in Supplementary File Figure S1. All signature scores are provided in Supplementary File Table S1.

Over 500 genes were examined and differential gene analysis between TIS^HIGH^ and TIS^LOW^ tumors revealed that CXCL9 (chemokine ligand 9), related to cytokine and chemokine signaling in the lymphoid compartment, and NKG7 (natural killer cell granule protein 7), related to cytotoxic granule exocytosis and inflammation, were significantly higher in TIS^HIGH^ tumors (log2 fold change of 5.77 and 3.84, respectively, corrected *p* = 0.0264 for both) when compared to TIS^LOW^ tumors (Figure 4B). Although nearly significant (*p* = 0.055), mRNA of CD8A (Cluster of Differentiation 8a), the cytotoxic T cell surface glycoprotein and GZMA (granzyme A), produced by CD8 T cells, were also upregulated 3.53- and 3.62-fold, respectively. A full list of differentially expressed genes in TIS^HIGH^ vs. TIS^LOW^ tumors is provided in Supplementary File Table S2.

**Figure 5:**
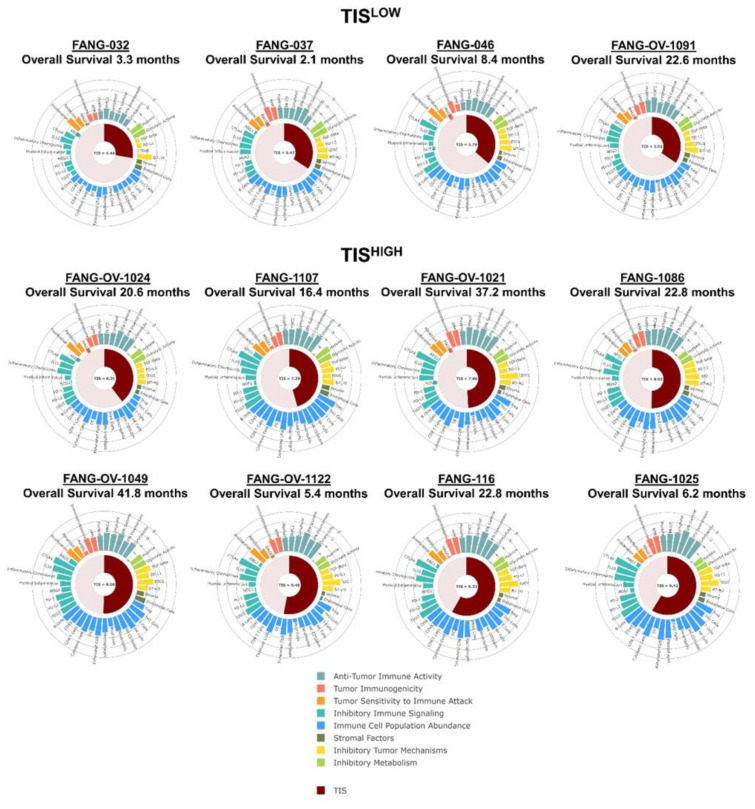
Patient tumor inflammation score (TIS) scores and relationship with survival and immune pathways.

**Figure 6:**
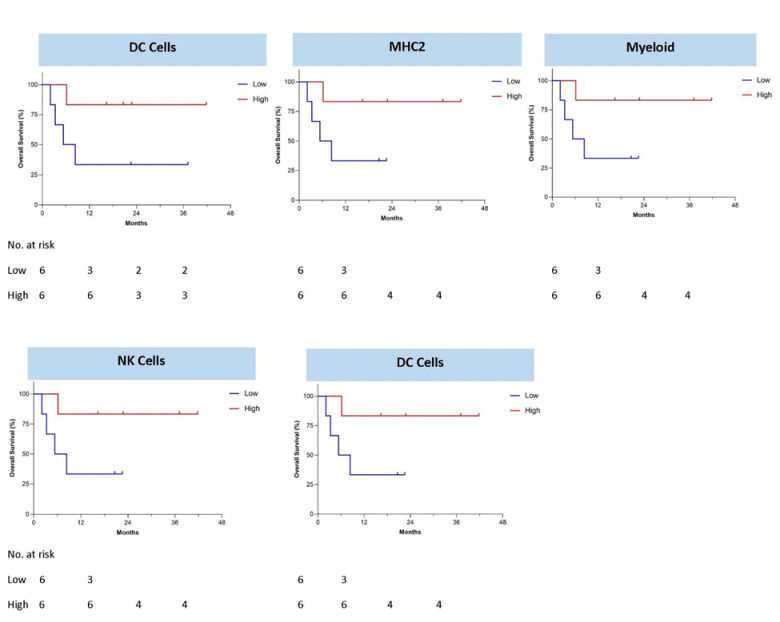
Baseline gene signatures correlate with overall survival after Vigil treatment.

**Figure 7:**
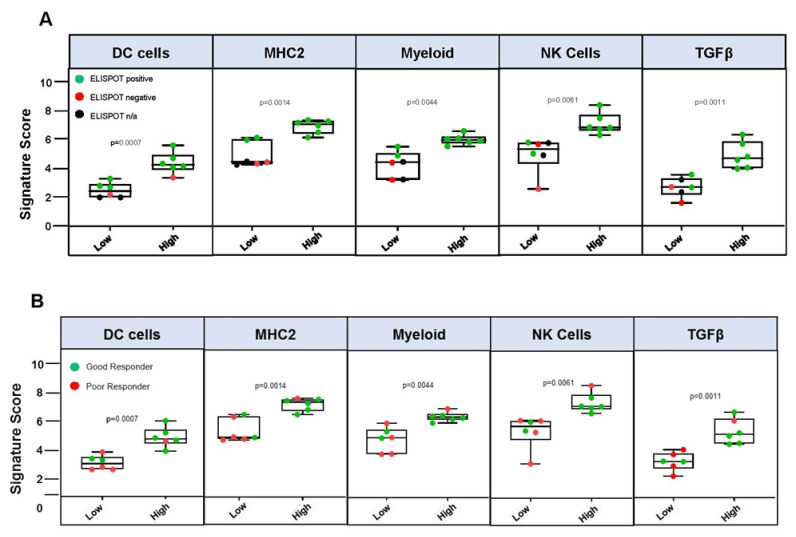
Box plots displaying the distribution of signature scores within the low- and high-expression groups (cutoff = score median) and correlation with (**A**) ELISPOT reactivity post-Vigil treatment. ELISPOT-positive (green), -negative (red) or not tested (black) patients are displayed. (**B**) Signature scores within the low- and high-expression groups (cutoff = score median) and correlation with overall survival > 12 months (GR) or <12 months (PR) post-Vigil treatment. GR (green), PR (red) patients are displayed. Whiskers represent minimum and maximum TIS scores. Statistical analyses of TIS scores were performed using unpaired T-tests with Welch correction.

OS was significantly improved in TIS^HIGH^ compared to TIS^LOW^ (median not reached vs. 5.8 months, one-sided log-rank *p* = 0.0379, log-rank HR 0.23 95% CI: 0.031–1.7) (Figure 4C). The 1-year OS rate was 25% versus 75%, respectively. Seven of eight (87.5%) TIS^HIGH^ patients demonstrated positive γ-IFN-ELISPOT reactivity after Vigil treatment compared to one of four (25%) TIS^LOW^ patients (Figure 4A). TIS score was significantly associated with γ-IFN-ELISPOT reactivity (*p* = 0.0002). Patient TIS relationship with survival varied based on the specific immune pathways that were important for each patient’s adaptive immune signature and could be important for potential immunotherapy targets (Figure 5). High MHC-II, dendritic cell (DC), myeloid, natural killer (NK) cells and TGFβ gene expression pre-Vigil treatment were all correlated with significantly longer OS (*p* = 0.038) (Figure 6). Notably, six patients (FANG-OV-1021, -1107, -116, -1086, -1025, 1049) displayed high MHC-II, myeloid, NK cells and TGFβ gene signatures prior to Vigil treatment and were γ-IFN-ELISPOT-positive and good responders (OS > 12mo) post-treatment (Figure 7A,B).

## 4. Discussion

OS assessment at 3 years suggested an advantage in a disparate group of solid tumor patients receiving Vigil. Continued evidence of OS advantage of more than 6 years was demonstrated in a homogenous group of recurrent/refractory ovarian cancer patients treated with Vigil who had a positive γ-IFN-ELISPOT response. In patients with similar treatment history, median OS at each recurrence has historically been reduced (i.e., first recurrence 17.6 months vs. fourth recurrence 6.2 months) [28]. Currently, there are several treatment options available for relapsed ovarian cancer, including platinum doublet regimens, single-agent chemotherapy (such as pegylated liposomal doxorubicin and topotecan), bevacizumab with or without chemotherapy and olaparib. None of these agents have demonstrated an advantage in relapse-free or overall survival. We did not find a difference in the number of lines of therapy between groups. Despite the small number of patients, the durability of the good response in the immune-activated (γ-IFN-ELISPOT) patients could be supportive of long-term memory stimulation by Vigil [14].

γ-IFN is a key modulator of cell-mediated immunity and controls the fate of T cells to undergo apoptosis or differentiate into memory T cells [29]. Tumor neoantigens presented by dendritic cells to naïve CD8+ T cells serve as a trigger for CD8+ T cells’ differentiation into cytotoxic T lymphocytes. Dendritic cells also activate CD4+ T helper cells through cross-presentation, which is essential for CD8+ T cell activation. Tumor antigen presentation by MHC class I and II molecules is critical to CD8+ and CD4+ T cell-mediated adaptive immune responses [30]. However, escape mechanisms related to this effect have evolved, including a decreased number and infiltration of T cells into the tumor microenvironment and the exhaustion of dysfunctional T cells [31,32]. Overcoming these deficiencies, converting cold into hot tumors, allows for reactivation of the immune system and antitumor control. Research has focused on increasing the number of T cells within the tumor microenvironment while also priming them to the individual tumor neoantigens to optimize the antitumor immune response. Increased levels of tumor-infiltrating lymphocytes (TILs) have been correlated with improved clinical responses in a variety of cancers, including ovarian cancer [33,34,35]. Therefore, several strategies have been employed to increase the number of TILs, including CAR-T cell therapy and vaccination. CAR-T cells modify the patients’ T cells to express targeted receptors to individual antigens. This approach has been successful in treating several hematologic malignancies, including acute lymphoblastic leukemia and large B cell lymphoma [36], although limited with most solid tumors [37]. Vaccination that presents the relevant tumor-specific neoantigens to dendritic cells via MHC-I and MHC-II, thus priming and expanding CD8+ T cells, is also an attractive therapeutic strategy. In this case, γ-IFN-ELISPOT response coupled with improved OS outcomes supports the hypothesis that Vigil promotes the production of memory T cells. In addition, Vigil use may relate to the education of T cells and other immune effector cells, as suggested by GEP analysis. This effect may be further enhanced in *BRCA* wild-type expressive malignancies [38] and may be relevant to the increased clonal neoantigen expression of the tumor, thereby providing more a comprehensive, long-term, antitumor, immune-targeting effect [39].

Opportunities for biomarker-associated Vigil sensitivity related to relevant signal pathway profiles are worth further exploration. Recently, a Phase IIb trial of newly diagnosed patients with stage III/IV ovarian cancer demonstrated greater clinical benefit involving significant advantages in RFS and OS in patients with *BRCA1/2* wild-type ovarian cancer [19]. These tumors largely have intact homologous recombination machinery, which hypothetically would result in higher expression of clonal neoantigens compared to *BRCA1/2* mutant tumors in which DNA repair is maximally disrupted [38,39].

Although y-IFN-related ELISPOT predicted the clinical response to immunotherapy, its results lack specificity, with an overlap between responsive and non-responsive cancers. Furthermore, it lacks the specificity to determine the potential mechanisms of the immune response, which would be the first step in determining a robust predictive biomarker for response. To tease out these subtle differences in immune factors, we performed mRNA gene expression profiling using the NanoString PanCancer IO360^TM^ panel on baseline tumor samples of patients that had received Vigil. This technology can digitally count up to 770 unique genes involving cancer cells, microenvironment and immune response. These data are easily translated into immune signatures to determine relevant clinical endpoints of response and survival. Additionally, a TIS incorporates an algorithm of 18 specific functional genes known to be associated with IFN-γ expression, which upregulates PD-L1 signaling and other immune modulators [40]. PD-L1 expression is a known biomarker of the response to checkpoint inhibitors that target PD-1, including pembrolizumab [41]. Therefore, high TIS is correlated with the response to immunotherapy [27] in a variety of cancers, particularly renal clear cell carcinoma [42], melanoma [43], lung [44] and head and neck tumors [45]. TIS determines the presence of a pre-existing, peripherally suppressed, adaptive immune response and “hot” vs. “cold” tumors by evaluating the expression of IFN, T-cell exhaustion, natural killer (NK) cells and antigen-presenting-cell-associated genes, such as MHC class I and II [40].

Danaher et al. reported TIS scores across a broad spectrum of cancer types, including ovarian cancer, serving as a pan-cancer measurement of the inflamed tumor [27]. While median TIS scores are higher in tumor types with higher response rates to immunomodulating therapies, within each tumor type, there is considerable inter-sample variability, limiting the applicability of most gene expression algorithms across tumor types. In contrast, because TIS depends primarily on genes expressed by immune cells or in response to immune signaling, it is plausible that its genes’ expression levels are driven by the magnitude of a tumor’s immune response and not by its cell of origin [40].

We demonstrated in this study the ability of TIS to detect “hot” ovarian cancer tumors, with a significant correlation of TIS^HIGH^ tumors with γ-IFN-ELISPOT positivity and prolonged OS. Additionally, Vigil-naïve tumors with high numbers of dendritic cells and MHC-II, as well as NK cells, demonstrated extended OS in patients post-Vigil treatment. TIS^HIGH^ tumors demonstrated significantly high mRNA expression of the cytokine and chemokine signaling gene CXCL9 as well as the regulator of NK cell exocytosis gene NKG7. The abundance of antigen-presenting cells and therefore the enhanced expression of MHC-II in treatment-naïve tumors may play a vital role in responsiveness to Vigil, which simultaneously increases MHC-dependent neoantigen presentation and DC maturation through GM-CSF expression. Additionally, tumors of patients with recurrent ovarian cancer showed higher TGFβ gene signaling in correlation with prolonged survival in response to Vigil. Previous studies have also demonstrated high levels of TGFβ expression in ovarian cancer cells, which is a core driver of T regulatory cell signaling and immunosuppression [46,47,48,49]. The Vigil plasmid is constructed to silence the expression of furin and downstream TGFβ1/2. Thus, tumor types with high TGFβ expression or gene signal pathways may increase responsiveness to Vigil treatment, as demonstrated here. The combination of TIS^HIGH^, MHC-II, high DC, NK cell and TGFβ signal pathway scores in recurrent ovarian cancer cells appear to be a likely driver of Vigil’s specificity to tumor specific neoantigens.

The safety and efficacy of Vigil would support its combination with other therapies with synergistic mechanisms, including checkpoint inhibitors and bevacizumab. Checkpoint inhibitors prevent the interaction between receptor (PD-1 or CTLA-4) and ligand (PD-L1 or CD80/86) in order to reactivate exhausted and expand antigen-specific T cells [50]. Bevacizumab also exerts immune effects, through the regulation of VEGF. VEGF blockade results in increased levels of CD4+ and CD8+ T cells combined with the downregulation of T regulatory cells [51,52]. Vigil would work in concert with these mechanisms to prime T cells as well as increase CD8+ T cell activity and decrease immune suppression [14]. Another future direction would include stratifying results based on homologous recombination status, either deficient or proficient, which may alter prognosis.

## 5. Conclusions

These results further support the safety and mechanism related to the efficacy of Vigil in ovarian cancer. This work was hypothesis-generating; however, Vigil’s unique dual immune stimulatory mechanism supported by γ-IFN-ELISPOT testing, coupled with vaccination to promote T cell priming, warrants continued further investigation in a larger cohort of ovarian cancer patients. TIS and its associated pathways hold promise in the discovery of biomarkers to predict the durable responses seen in this 6-year long-term follow-up of recurrent ovarian cancer patients that received Vigil. Biomarker determination via molecular profile assessment and/or NanoString^®^-based characterization is ongoing to define specific populations of patients with cancer and *BRCA1/2* wild-type gene expression for evidence of further sensitivity or resistance to Vigil.

## 6. Summary Points

- OS at 6 years from tissue procurement was 58%.
- OS benefit was observed in γ-IFN ELISPOT-positive response (36.8 vs. 23.0 months HR 0.19, *p* = 0.0098).
- TIS^HIGH^ compared to TIS^LOW^ demonstrated OS benefit to Vigil treatment (1-year OS 75 vs. 25% *p* = 0.03795).
- Correlated survival benefit of Vigil induced immune response via ELISPOT and relevant indication (TIS > 6, MHC II) using NanoString.

## Figures and Tables

**Table 1:** Patient demographics.

	Recurrent Ovarian Cancer Patients	NanoString^®^ Analysis Recurrent Ovarian Cancer Patients
Patients—no.	21	12
Age—years		
Median	61	61.5
Mean	59.8	59.6
Range	39–75	39–75
<65—no. (%)	16 (76.2)	10 (83.3)
≥65—no. (%)	5 (23.8)	2 (16.7)
No. of prior lines—no. (%)		
Median	2	2
Mean	2.95	3.17
Range	1–10	1–10
CA-125 at time of treatment start—no. (%)		
Median	17.4	17.3
Mean	150.4	170.1
Range	17.4–1434	8.8–1434
<35	11 (52.4)	8 (66.7)
≥35	4 (19.0)	2 (16.7)
Missing	6 (28.6)	2 (16.7)
Disease at study start—no. (%)		
No disease	3 (14.3) *	2 (16.7) *
disease	18 (85.7)	10 (83.3)

* Patients had no evidence of disease (NED) (* two subjects with elevated CA-125, one subject with no visible disease by RECIST after 3rd line chemotherapy) prior to therapy for recurrence before enrolling in trial.

## Data Availability

Study data maybe shared upon request and after approval of a data sharing proposal. Proposals that pose a conflict of interest or competitive risk might be declined by Gradalis.

## Supplementary Materials

The following are available online at https://www.mdpi.com/article/10.3390/vaccines9080894/s1, Figure S1: Heat map of twenty-seven significant signature scores and Principal Component Analysis, Table S1: Immune pathway results, Table S2: Gene expression analysis.

## Abbreviations

OS	overall survival
GEP	gene expression profile
TIS	tumor inflammation signature
MHC	major histocompatibility class
DC	dendritic cells
NK	natural killer
PARP	poly (ADP-ribose) polymerase
PCA	principal component analysis
GR	good response
