# Supplementary material for: Long-Term Follow-Up of Gemogenovatucel-T (Vigil) Survival and Molecular Signals of Immune Response in Recurrent Ovarian Cancer
Source: Vaccines (Basel). 2021 Aug 12;9(8):894. doi: 10.3390/vaccines9080894 (PMC8402348; doi:10.3390/vaccines9080894)

Supplemental Figure S1. Heat map of twenty-seven significant signatures scores and Principal Component Analysis.

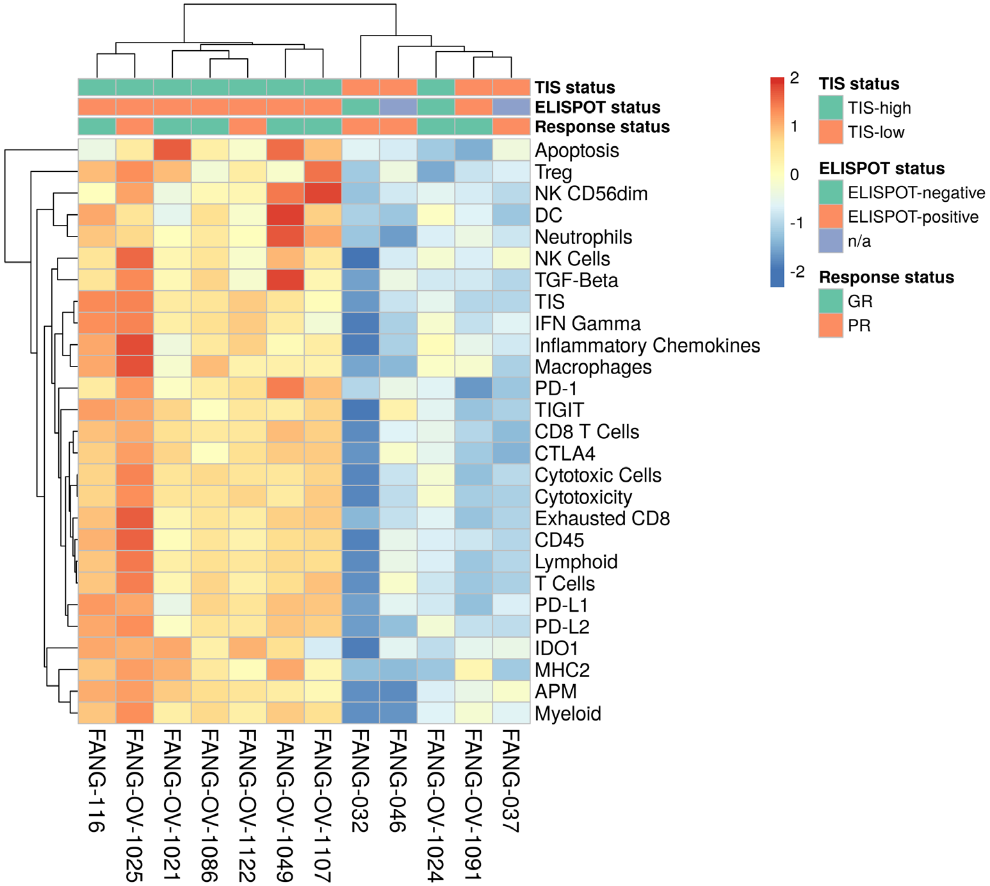

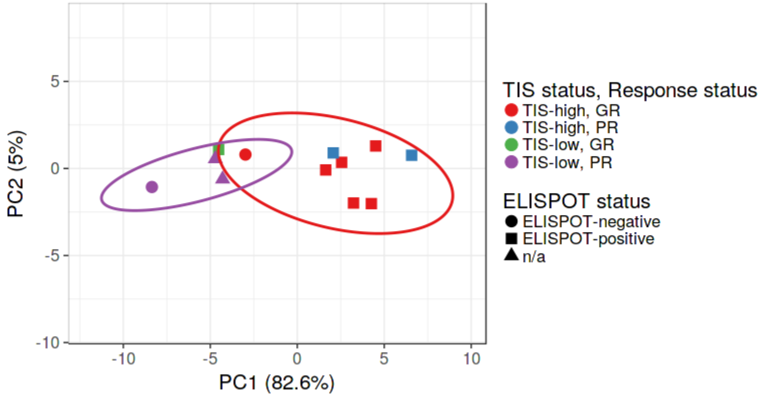

Supplement: Supplementary file 1 [file vaccines-09-00894-s001.zip › vaccines-1279453 after proof figures.pdf]
