# Supplementary material for: Long-Term Follow-Up of Gemogenovatucel-T (Vigil) Survival and Molecular Signals of Immune Response in Recurrent Ovarian Cancer
Source: Vaccines (Basel). 2021 Aug 12;9(8):894. doi: 10.3390/vaccines9080894 (PMC8402348; doi:10.3390/vaccines9080894)
Supplement: Supplementary file 1 [file vaccines-09-00894-s001.zip › vaccines-1279453 after proof non publ.pdf]

Supplemental File 2. Table S2. Gene Expression Analysis

	Log2 fold change	std error (log2)	Lower confidence limit (log2)
CXCL9-mRNA	5.77	0.625	4.55
NKG7-mRNA	3.84	0.453	2.96
CD8A-mRNA	3.53	0.495	2.56
GZMA-mRNA	3.62	0.511	2.62
CCL5-mRNA	3.53	0.549	2.46
GBP4-mRNA	3.82	0.598	2.65
IFNG-mRNA	4.18	0.676	2.85
CTSW-mRNA	3.57	0.625	2.34
CXCL13-mRNA	4.92	0.865	3.22
IER3-mRNA	2.18	0.387	1.42
IL2RB-mRNA	4.22	0.751	2.75
LYZ-mRNA	2.78	0.496	1.8
RUNX3-mRNA	2.19	0.4	1.41
KLRK1-mRNA	3.97	0.731	2.53
GIMAP4-mRNA	3.26	0.602	2.08
KLRB1-mRNA	3.38	0.628	2.15
CCR5-mRNA	3.08	0.578	1.94
SAMSN1-mRNA	2.53	0.48	1.59
CXCR6-mRNA	4.47	0.858	2.79
KLRD1-mRNA	4.44	0.862	2.75
GIMAP6-mRNA	4.34	0.843	2.69
CD3D-mRNA	3.47	0.677	2.14
PDCD1LG2-mRNA	3.79	0.745	2.33
TLR4-mRNA	3.07	0.605	1.88
GBP1-mRNA	2.59	0.519	1.58
BAX-mRNA	1.54	0.308	0.932
C1QB-mRNA	3.51	0.707	2.13
IL10RA-mRNA	2.69	0.547	1.62
IL2RG-mRNA	3.39	0.689	2.04
GZMH-mRNA	4.16	0.849	2.5
CD96-mRNA	3.44	0.703	2.06
TYMP-mRNA	2.57	0.526	1.53
CXCL10-mRNA	3.68	0.757	2.2
IL32-mRNA	2.57	0.53	1.53
CD2-mRNA	2.92	0.607	1.73
ITGAL-mRNA	3.27	0.684	1.93
HCK-mRNA	2.67	0.558	1.57
CTLA4-mRNA	3.77	0.797	2.2
LY96-mRNA	2.34	0.497	1.37
CXCL11-mRNA	4.39	0.934	2.56
FASLG-mRNA	3.75	0.8	2.18
CTSS-mRNA	2.13	0.457	1.24
JAK2-mRNA	2.22	0.476	1.28
CYBB-mRNA	2.7	0.581	1.56
GZMK-mRNA	3.54	0.762	2.05
PIK3CG-mRNA	2.83	0.61	1.64

CD3G-mRNA	4.16	0.897	2.4
LAG3-mRNA	4.35	0.938	2.51
IRF4-mRNA	4.39	0.956	2.51
PTPRC-mRNA	2.69	0.586	1.54
TNFRSF9-mRNA	3.04	0.665	1.73
RAD51C-mRNA	-1.51	0.331	-2.15
LILRB2-mRNA	3.03	0.671	1.71
ITGAX-mRNA	2.12	0.471	1.19
PFKM-mRNA	-1.51	0.337	-2.17
C1QA-mRNA	2.98	0.667	1.68
CD38-mRNA	4.31	0.971	2.4
CD45RO-mRNA	2.5	0.564	1.39
CCL2-mRNA	1.89	0.427	1.06
STAT1-mRNA	2.06	0.466	1.15
ZEB2-mRNA	2.19	0.495	1.21
DAB2-mRNA	2.14	0.486	1.19
LCK-mRNA	3.17	0.724	1.75
ENTPD1-mRNA	2.34	0.548	1.27
CCND2-mRNA	2.29	0.535	1.24
FCN1-mRNA	2.41	0.568	1.29
CASP1-mRNA	2.36	0.559	1.27
CD68-mRNA	2.26	0.537	1.21
PECAM1-mRNA	2.23	0.533	1.19
TNFRSF1B-mRNA	2.49	0.595	1.32
CLECL1-mRNA	3.32	0.8	1.75
FPR3-mRNA	2.48	0.598	1.31
GLUL-mRNA	1.2	0.294	0.624
LILRB4-mRNA	2.56	0.63	1.32
B2M-mRNA	1.99	0.492	1.02
ICOS-mRNA	3.79	0.94	1.95
FCGR1A-mRNA	2.38	0.593	1.22
IRF8-mRNA	2.9	0.724	1.48
CD84-mRNA	2.26	0.568	1.15
SERPINH1-mRNA	-1.5	0.378	-2.25
CD28-mRNA	2.46	0.623	1.24
FBP1-mRNA	1.88	0.476	0.947
TGFB1-mRNA	2.24	0.567	1.13
FCGR3A/B-mRNA	2.58	0.66	1.28
LAIR1-mRNA	2.44	0.629	1.21
GNLY-mRNA	3.32	0.86	1.64
ITGB2-mRNA	1.9	0.493	0.937
CD80-mRNA	3.36	0.87	1.65
MS4A6A-mRNA	3.64	0.948	1.79
ITGA4-mRNA	2.13	0.557	1.04
TIGIT-mRNA	3.57	0.933	1.74
CD3E-mRNA	3.39	0.899	1.62
PSMB9-mRNA	2.42	0.644	1.16

HLA-E-mRNA	1.68	0.449	0.8
HLA-DPB1-mRNA	2.48	0.664	1.18
IL16-mRNA	2.54	0.683	1.21
CD14-mRNA	2.08	0.559	0.982
CSF1R-mRNA	2.28	0.615	1.08
OAS2-mRNA	2.66	0.72	1.25
TREM2-mRNA	1.72	0.466	0.803
CDKN1A-mRNA	2.39	0.65	1.12
CD7-mRNA	3.07	0.837	1.43
OASL-mRNA	2.85	0.775	1.33
MSH6-mRNA	-0.988	0.269	-1.52
TNF-mRNA	2.27	0.62	1.05
GBP2-mRNA	2.2	0.602	1.02
TRAT1-mRNA	3.53	0.973	1.62
CD45RB-mRNA	2.27	0.628	1.04
CD27-mRNA	3.23	0.904	1.46
MS4A4A-mRNA	2.54	0.709	1.15
LILRA1-mRNA	2.47	0.69	1.11
HDAC5-mRNA	-1.16	0.327	-1.81
TAP1-mRNA	2.3	0.65	1.02
CCL4-mRNA	2.64	0.751	1.17
CD5-mRNA	2.67	0.76	1.18
HLA-B-mRNA	1.83	0.524	0.801
HLA-C-mRNA	1.5	0.433	0.651
VCAM1-mRNA	2.93	0.85	1.27
CD44-mRNA	1.51	0.437	0.651
CD300A-mRNA	1.93	0.564	0.827
TNFAIP3-mRNA	2.23	0.651	0.953
JAK3-mRNA	2.25	0.658	0.959
GPSM3-mRNA	2.12	0.622	0.905
TLR2-mRNA	1.96	0.574	0.832
STAT4-mRNA	3.29	0.966	1.4
BBS1-mRNA	-1.13	0.333	-1.78
CCR4-mRNA	3.31	0.993	1.36
TRAF1-mRNA	1.81	0.544	0.744
PIK3CD-mRNA	1.52	0.46	0.622
CCL8-mRNA	3.68	1.12	1.49
PIK3R5-mRNA	2.12	0.645	0.86
CD69-mRNA	2.74	0.832	1.11
HAVCR2-mRNA	2.14	0.658	0.854
EOMES-mRNA	2.87	0.884	1.14
CCL3/L1-mRNA	2.51	0.776	0.993
ITGAM-mRNA	2.01	0.62	0.791
TAP2-mRNA	1.79	0.555	0.703
CD48-mRNA	2.51	0.784	0.971
FCGR2A-mRNA	1.69	0.53	0.651
ZAP70-mRNA	3.15	0.99	1.21

HLA-A-mRNA	2.66	0.845	1.01
CD86-mRNA	2.4	0.763	0.909
SH2D1A-mRNA	3.14	0.997	1.19
LTB-mRNA	2.67	0.851	0.999
TBXAS1-mRNA	1.74	0.558	0.645
NCR1-mRNA	2.42	0.783	0.885
APOE-mRNA	1.76	0.572	0.64
SOCS1-mRNA	2.32	0.756	0.839
IL11RA-mRNA	-1.84	0.599	-3.01
HLA-DRB1-mRNA	1.9	0.622	0.678
CMKLR1-mRNA	3.15	1.03	1.12
PC-mRNA	-1.37	0.449	-2.25
HLA-F-mRNA	2.22	0.735	0.78
HLA-DPA1-mRNA	2.21	0.733	0.769
LY9-mRNA	3.43	1.14	1.19
OAS1-mRNA	2.05	0.684	0.712
PTGER4-mRNA	2.05	0.683	0.709
NLRC5-mRNA	1.98	0.662	0.687
CLEC7A-mRNA	2.24	0.748	0.77
CD163-mRNA	1.9	0.635	0.653
IFI35-mRNA	1.88	0.632	0.637
PVRIG-mRNA	2.25	0.758	0.765
IRF1-mRNA	1.6	0.549	0.526
FCGR2B-mRNA	1.46	0.508	0.46
IL7R-mRNA	2.87	1.02	0.884
CD74-mRNA	1.74	0.623	0.519
ALDOC-mRNA	-1.29	0.464	-2.2
ISG15-mRNA	2.12	0.766	0.614
RELB-mRNA	0.857	0.31	0.248
MX1-mRNA	2.2	0.804	0.622
MAP3K12-mRNA	-1.36	0.501	-2.34
HLA-DMB-mRNA	1.73	0.648	0.463
P2RY13-mRNA	3.17	1.19	0.835
IRF7-mRNA	1.82	0.688	0.477
IRF2-mRNA	0.8	0.302	0.207
IRF3-mRNA	0.701	0.266	0.18
OLR1-mRNA	1.87	0.71	0.479
SREBF1-mRNA	-1.02	0.387	-1.78
BCL2-mRNA	1.64	0.624	0.416
OAS3-mRNA	1.77	0.675	0.443
TLR1-mRNA	2.46	0.944	0.609
VTCN1-mRNA	-1.92	0.737	-3.36
BRD3-mRNA	-0.684	0.263	-1.2
FYN-mRNA	0.988	0.38	0.243
BBC3-mRNA	1.55	0.596	0.38
CD4-mRNA	2.21	0.857	0.53
ITGA1-mRNA	1.15	0.445	0.273

A2M-mRNA	1.28	0.497	0.302
TNFSF8-mRNA	2.1	0.822	0.493
SIGLEC1-mRNA	2.82	1.1	0.659
CCR2-mRNA	2.23	0.872	0.519
MFNG-mRNA	1.33	0.523	0.302
SIRPB2-mRNA	1.77	0.702	0.391
RORC-mRNA	-1.61	0.645	-2.87
LGALS9-mRNA	1.46	0.592	0.299
AXL-mRNA	1.85	0.751	0.379
TLR7-mRNA	2.24	0.913	0.448
HMGB1-mRNA	-1.02	0.417	-1.84
MTOR-mRNA	-0.389	0.159	-0.701
RPTOR-mRNA	-1.13	0.466	-2.04
TNFSF13B-mRNA	1.92	0.796	0.362
MRC1-mRNA	2.22	0.928	0.401
CX3CR1-mRNA	2.24	0.939	0.402
TMEM140-mRNA	2.17	0.914	0.383
MYD88-mRNA	0.958	0.403	0.168
RELA-mRNA	-0.559	0.235	-1.02
EIF2B4-mRNA	-0.837	0.352	-1.53
FADD-mRNA	-0.463	0.196	-0.847
SYK-mRNA	0.804	0.34	0.138
IL1B-mRNA	2.06	0.875	0.349
IRF5-mRNA	0.998	0.428	0.16
CASP9-mRNA	-0.495	0.212	-0.911
CDKN2A-mRNA	1.37	0.59	0.216
APOL6-mRNA	1.26	0.541	0.196
XCL1/2-mRNA	1.76	0.761	0.269
CDK6-mRNA	-1.27	0.549	-2.35
SMAD5-mRNA	-0.805	0.351	-1.49
MMP9-mRNA	1.48	0.649	0.209
PRKCA-mRNA	-1.62	0.709	-3.01
RSAD2-mRNA	1.94	0.854	0.266
MMRN2-mRNA	-1.53	0.679	-2.86
IFIT2-mRNA	1.88	0.841	0.229
NFKBIA-mRNA	1.17	0.529	0.136
CSF1-mRNA	1.26	0.57	0.147
NFATC2-mRNA	1.09	0.491	0.124
DNMT1-mRNA	0.931	0.423	0.102
MAP3K7-mRNA	-0.455	0.207	-0.861
SNCA-mRNA	-1.66	0.757	-3.14
IFIH1-mRNA	1.5	0.686	0.152
GLS-mRNA	1.15	0.529	0.11
WNT5A-mRNA	1.94	0.927	0.122
MS4A1-mRNA	2.46	1.18	0.145
PSMB8-mRNA	1.13	0.542	0.0632
NOD2-mRNA	1.32	0.634	0.0731

CSF3R-mRNA	1.43	0.695	0.0633
FPR1-mRNA	1.75	0.855	0.077
THY1-mRNA	-2.03	0.993	-3.98
MSH2-mRNA	-0.8	0.391	-1.57
ARNT2-mRNA	-1.99	0.975	-3.9
ICAM3-mRNA	0.912	0.451	0.0286
SERPINA1-mRNA	1.64	0.81	0.0505
SBNO2-mRNA	0.807	0.4	0.0228
IFI27-mRNA	1.42	0.703	0.0396
MRE11-mRNA	-0.631	0.314	-1.25
PIK3R2-mRNA	-0.822	0.41	-1.63
HLA-DRA-mRNA	1.36	0.682	0.0232
CCND3-mRNA	0.897	0.452	0.0117
GOT2-mRNA	-0.517	0.262	-1.03
PARP9-mRNA	1.02	0.52	0.0028
SAMD9-mRNA	1.28	0.654	0.00118
RPS6KB1-mRNA	-0.589	0.301	-1.18
VSIR-mRNA	1.1	0.566	-0.00872
MFGE8-mRNA	-1.56	0.804	-3.14
PALMD-mRNA	-1.96	1.01	-3.95
HLA-DMA-mRNA	1.39	0.714	-0.0142
FCGRT-mRNA	0.805	0.416	-0.0113
IL6-mRNA	2.07	1.09	-0.0678
DUSP2-mRNA	1.25	0.663	-0.0451
VEGFB-mRNA	-0.574	0.304	-1.17
IFIT3-mRNA	1.51	0.803	-0.0597
MET-mRNA	-1.21	0.648	-2.48
SGK1-mRNA	1.13	0.605	-0.0587
CD79A-mRNA	3.41	1.85	-0.221
ICOSLG-mRNA	0.733	0.401	-0.0522
PROM1-mRNA	1.9	1.05	-0.156
TNFRSF11A-mRNA	1.59	0.887	-0.144
PARP12-mRNA	0.775	0.432	-0.0712
IL1RN-mRNA	1.63	0.908	-0.152
RB1-mRNA	0.705	0.394	-0.0667
PSMB5-mRNA	-0.431	0.241	-0.904
CD19-mRNA	1.93	1.08	-0.194
IDO1-mRNA	3.49	1.98	-0.387
BAD-mRNA	-0.514	0.292	-1.09
PRKAA2-mRNA	-1.33	0.76	-2.82
TNFSF12-mRNA	0.791	0.454	-0.0976
P4HA1-mRNA	-0.814	0.467	-1.73
NOTCH1-mRNA	-0.996	0.578	-2.13
TAPBP-mRNA	0.816	0.475	-0.115
IL15-mRNA	0.928	0.541	-0.133
TCF3-mRNA	0.5	0.293	-0.0733
NECTIN1-mRNA	-1.37	0.799	-2.93

NFIL3-mRNA	-0.904	0.529	-1.94
CCNA1-mRNA	1.6	0.946	-0.251
CD276-mRNA	-0.595	0.351	-1.28
CLEC5A-mRNA	1.2	0.724	-0.22
MICA-mRNA	-1.09	0.66	-2.38
PRKACB-mRNA	0.993	0.601	-0.186
CCL18-mRNA	0.986	0.603	-0.197
PSMB10-mRNA	1.12	0.696	-0.239
SLC16A1-mRNA	-1.32	0.818	-2.92
IFI6-mRNA	1.19	0.746	-0.276
IFNGR2-mRNA	0.403	0.258	-0.103
ERBB2-mRNA	-0.688	0.442	-1.55
OLFML2B-mRNA	1.35	0.87	-0.355
CDKN2B-mRNA	0.892	0.58	-0.245
GOT1-mRNA	-0.54	0.353	-1.23
DTX4-mRNA	-1.34	0.877	-3.06
IFIT1-mRNA	1.23	0.815	-0.366
KRAS-mRNA	-0.386	0.257	-0.889
DUSP5-mRNA	1.17	0.784	-0.366
RRM2-mRNA	-0.973	0.653	-2.25
WNT10A-mRNA	-1.55	1.05	-3.61
VEGFA-mRNA	-1.21	0.819	-2.82
DTX3L-mRNA	0.646	0.437	-0.21
PVR-mRNA	-0.773	0.524	-1.8
FGFR1-mRNA	-1.07	0.732	-2.51
PMS2-mRNA	-0.348	0.24	-0.82
NOTCH2-mRNA	-0.568	0.392	-1.34
PTCD2-mRNA	-0.564	0.391	-1.33
MXI1-mRNA	-0.902	0.626	-2.13
CRABP2-mRNA	-1.31	0.912	-3.1
EIF2AK2-mRNA	0.664	0.467	-0.251
PKM-mRNA	-0.443	0.312	-1.05
S100A8-mRNA	0.965	0.68	-0.368
IRF9-mRNA	0.571	0.403	-0.22
MB21D1-mRNA	0.669	0.479	-0.269
NFKB2-mRNA	0.484	0.348	-0.197
ARID1A-mRNA	-0.252	0.181	-0.607
NF1-mRNA	-0.509	0.367	-1.23
FGF18-mRNA	-1.2	0.888	-2.94
ITGAV-mRNA	-0.622	0.46	-1.52
RPL7A-mRNA	-0.386	0.289	-0.953
ITPK1-mRNA	-0.261	0.196	-0.645
HIF1A-mRNA	0.377	0.284	-0.179
C2-mRNA	0.837	0.635	-0.406
PDZK1IP1-mRNA	-1.58	1.21	-3.94
SPP1-mRNA	1.07	0.819	-0.539
CEP55-mRNA	0.812	0.624	-0.411

HDAC11-mRNA	-0.759	0.587	-1.91
NBN-mRNA	-0.348	0.271	-0.879
EPCAM-mRNA	-0.666	0.518	-1.68
IFITM1-mRNA	0.878	0.697	-0.488
TPM1-mRNA	-0.607	0.482	-1.55
TP53-mRNA	0.787	0.627	-0.442
RIPK3-mRNA	0.674	0.537	-0.379
IFNAR1-mRNA	-0.461	0.368	-1.18
KAT2B-mRNA	0.571	0.461	-0.332
COL5A1-mRNA	-1.03	0.848	-2.7
IFI16-mRNA	0.879	0.729	-0.549
SLC7A5-mRNA	0.974	0.814	-0.622
TRIM21-mRNA	0.847	0.715	-0.555
IL18-mRNA	0.869	0.738	-0.577
COL4A5-mRNA	-1.19	1.01	-3.17
UBA7-mRNA	0.593	0.507	-0.402
CDC20-mRNA	0.772	0.663	-0.528
ITGB8-mRNA	-1.02	0.883	-2.76
NFKBIE-mRNA	0.569	0.493	-0.398
CTNNB1-mRNA	-0.302	0.262	-0.816
SNAI1-mRNA	0.548	0.476	-0.384
TGFBR1-mRNA	0.318	0.279	-0.228
SFXN1-mRNA	-0.393	0.345	-1.07
CDH11-mRNA	-0.952	0.84	-2.6
ITGA6-mRNA	-0.518	0.458	-1.42
CD58-mRNA	0.787	0.696	-0.577
ATM-mRNA	0.362	0.321	-0.267
PIAS4-mRNA	0.299	0.265	-0.221
PTEN-mRNA	-0.668	0.599	-1.84
PIK3CA-mRNA	-0.377	0.351	-1.07
TNFRSF14-mRNA	0.462	0.432	-0.384
CD36-mRNA	0.991	0.929	-0.829
EGFR-mRNA	-1.21	1.14	-3.45
MICB-mRNA	0.88	0.831	-0.748
HERC6-mRNA	0.864	0.82	-0.742
BNIP3-mRNA	0.63	0.599	-0.544
PDK1-mRNA	-0.696	0.662	-1.99
NCAM1-mRNA	-1.06	1.03	-3.09
HLA-DOA-mRNA	0.798	0.777	-0.726
IL1R2-mRNA	0.926	0.904	-0.846
STAT2-mRNA	0.442	0.434	-0.408
PNOC-mRNA	-0.679	0.667	-1.99
PLOD2-mRNA	-0.89	0.88	-2.61
CHUK-mRNA	0.376	0.373	-0.355
FLNB-mRNA	-0.548	0.557	-1.64
CX3CL1-mRNA	-0.845	0.86	-2.53
TGFBR2-mRNA	0.389	0.398	-0.392

ATF3-mRNA	0.863	0.899	-0.898
TGFB2-mRNA	1.03	1.07	-1.08
CXCR4-mRNA	0.542	0.567	-0.569
SLC11A1-mRNA	0.571	0.609	-0.624
MAP3K8-mRNA	0.373	0.399	-0.409
IKBKG-mRNA	0.486	0.521	-0.536
DUSP1-mRNA	0.651	0.699	-0.719
TNFRSF10B-mRNA	0.689	0.749	-0.779
RICTOR-mRNA	-0.323	0.351	-1.01
RASAL1-mRNA	-0.368	0.403	-1.16
EPM2AIP1-mRNA	-0.298	0.331	-0.947
TTC30A-mRNA	-0.537	0.608	-1.73
BRCA1-mRNA	-0.759	0.863	-2.45
ROCK1-mRNA	0.27	0.31	-0.338
BID-mRNA	-0.476	0.548	-1.55
IFITM2-mRNA	-0.478	0.554	-1.56
ITGAE-mRNA	0.189	0.22	-0.241
TNFSF4-mRNA	0.448	0.535	-0.602
MELK-mRNA	0.468	0.567	-0.644
TLR8-mRNA	-0.645	0.785	-2.18
BNIP3L-mRNA	0.402	0.493	-0.564
NRAS-mRNA	0.22	0.271	-0.311
TPSAB1/B2-mRNA	0.654	0.814	-0.941
TICAM1-mRNA	-0.565	0.704	-1.94
AXIN1-mRNA	0.476	0.595	-0.69
VCAN-mRNA	0.565	0.708	-0.823
MKI67-mRNA	0.442	0.558	-0.651
FUT4-mRNA	0.552	0.702	-0.823
IGF2R-mRNA	-0.303	0.386	-1.06
DDB2-mRNA	0.47	0.602	-0.71
FANCA-mRNA	-0.586	0.752	-2.06
GLUD1-mRNA	-0.207	0.267	-0.731
HDAC4-mRNA	-0.333	0.43	-1.18
E2F3-mRNA	-0.53	0.688	-1.88
NEIL1-mRNA	-0.647	0.846	-2.31
RIPK2-mRNA	0.374	0.496	-0.597
DPP4-mRNA	-0.539	0.725	-1.96
RBL2-mRNA	-0.249	0.342	-0.919
F2RL1-mRNA	-0.668	0.932	-2.49
TNFRSF1A-mRNA	-0.234	0.329	-0.878
TNFSF10-mRNA	0.609	0.885	-1.12
NECTIN2-mRNA	-0.283	0.414	-1.09
IL6R-mRNA	0.249	0.366	-0.469
BRIP1-mRNA	-0.429	0.637	-1.68
SRP54-mRNA	-0.145	0.217	-0.57
ESR1-mRNA	-0.372	0.556	-1.46
PCK2-mRNA	0.252	0.39	-0.512

PPARGC1B-mRNA	-0.433	0.673	-1.75
CASP8-mRNA	-0.346	0.548	-1.42
CDH2-mRNA	-0.756	1.2	-3.1
RAD50-mRNA	-0.158	0.254	-0.655
TMEM173-mRNA	0.402	0.649	-0.869
ELOB-mRNA	-0.45	0.728	-1.88
WNT2B-mRNA	0.384	0.625	-0.84
HELLS-mRNA	-0.301	0.495	-1.27
HDAC3-mRNA	-0.145	0.246	-0.628
ROR2-mRNA	-0.625	1.06	-2.7
BRCA2-mRNA	0.45	0.768	-1.06
MGMT-mRNA	0.355	0.609	-0.839
ERO1A-mRNA	-0.329	0.569	-1.44
LTBP1-mRNA	-0.586	1.03	-2.6
FZD8-mRNA	-0.486	0.868	-2.19
SMAP1-mRNA	-0.134	0.24	-0.603
TWF1-mRNA	-0.196	0.361	-0.904
GPC4-mRNA	0.458	0.848	-1.2
JAK1-mRNA	0.184	0.347	-0.497
EIF4EBP1-mRNA	-0.217	0.414	-1.03
IKBKB-mRNA	0.304	0.583	-0.838
ANGPTL4-mRNA	-1.08	2.08	-5.16
AKT1-mRNA	0.143	0.28	-0.406
ICAM1-mRNA	-0.411	0.822	-2.02
MAML2-mRNA	-0.256	0.519	-1.27
MMP7-mRNA	-0.816	1.67	-4.09
APH1B-mRNA	-0.332	0.684	-1.67
ZC3H12A-mRNA	0.285	0.586	-0.864
CXCL8-mRNA	0.367	0.763	-1.13
EXO1-mRNA	-0.426	0.888	-2.17
TPI1-mRNA	-0.191	0.403	-0.982
SLC1A5-mRNA	-0.182	0.384	-0.935
WDR76-mRNA	0.204	0.431	-0.641
P4HA2-mRNA	-0.208	0.452	-1.09
NFKB1-mRNA	0.238	0.521	-0.783
HRAS-mRNA	-0.235	0.516	-1.25
RAD51-mRNA	-0.31	0.687	-1.66
PARP4-mRNA	-0.143	0.317	-0.765
FAS-mRNA	-0.389	0.867	-2.09
HES1-mRNA	-0.29	0.668	-1.6
CASP3-mRNA	0.153	0.356	-0.545
ALDOA-mRNA	-0.17	0.399	-0.953
LAMB3-mRNA	-0.334	0.787	-1.88
CDKN1C-mRNA	-0.174	0.414	-0.986
SLC2A1-mRNA	-0.302	0.72	-1.71
CDH1-mRNA	-0.269	0.646	-1.54
GPR160-mRNA	-0.289	0.719	-1.7

COL6A3-mRNA	-0.33	0.833	-1.96
KIF2C-mRNA	0.324	0.82	-1.28
CEBPB-mRNA	-0.151	0.384	-0.902
BIRC5-mRNA	-0.214	0.552	-1.3
ZEB1-mRNA	-0.333	0.879	-2.06
ANLN-mRNA	-0.267	0.736	-1.71
H2AFX-mRNA	-0.13	0.358	-0.831
CXCL3-mRNA	-0.327	0.903	-2.1
MYC-mRNA	-0.308	0.857	-1.99
TREM1-mRNA	-0.272	0.758	-1.76
AREG-mRNA	-0.644	1.8	-4.18
RPL23-mRNA	0.113	0.318	-0.51
PFKFB3-mRNA	0.217	0.62	-0.998
LDHB-mRNA	-0.124	0.359	-0.828
TNFRSF25-mRNA	0.235	0.69	-1.12
HLA-DOB-mRNA	-0.404	1.2	-2.76
ADM-mRNA	-0.262	0.782	-1.79
CDK2-mRNA	-0.113	0.337	-0.774
ID4-mRNA	-0.325	0.982	-2.25
WNT11-mRNA	0.359	1.16	-1.91
HLA-DQA1-mRNA	1.01	3.28	-5.42
CCNB1-mRNA	0.161	0.534	-0.885
CXCL2-mRNA	0.255	0.848	-1.41
CENPF-mRNA	0.24	0.802	-1.33
MLH1-mRNA	0.0659	0.223	-0.371
STAT3-mRNA	-0.0933	0.337	-0.754
IFNGR1-mRNA	0.139	0.514	-0.868
PIK3R1-mRNA	0.12	0.474	-0.808
TLR5-mRNA	-0.119	0.471	-1.04
HLA-DQB1-mRNA	0.635	2.6	-4.46
BCL2L1-mRNA	-0.0784	0.324	-0.714
BRD4-mRNA	-0.09	0.373	-0.822
FSTL3-mRNA	-0.27	1.12	-2.47
NT5E-mRNA	-0.184	0.773	-1.7
VHL-mRNA	-0.064	0.283	-0.618
ARG2-mRNA	-0.138	0.61	-1.33
S100A9-mRNA	0.198	0.876	-1.52
CD40-mRNA	0.14	0.62	-1.08
GAS1-mRNA	-0.211	0.945	-2.06
LDHA-mRNA	-0.1	0.493	-1.07
EZH2-mRNA	-0.06	0.311	-0.67
TNFRSF11B-mRNA	-0.215	1.11	-2.4
MAP3K5-mRNA	0.0937	0.502	-0.891
CCL20-mRNA	-0.229	1.26	-2.69
TAPBPL-mRNA	-0.0953	0.568	-1.21
FLT1-mRNA	-0.133	0.796	-1.69
THBS1-mRNA	0.142	0.869	-1.56

CXCL16-mRNA	0.0988	0.609	-1.09
API5-mRNA	-0.0355	0.235	-0.496
RNLS-mRNA	-0.118	0.787	-1.66
CBLC-mRNA	0.121	0.863	-1.57
TNKS-mRNA	-0.0668	0.501	-1.05
MARCO-mRNA	0.134	1.01	-1.84
PGPEP1-mRNA	-0.0584	0.542	-1.12
APC-mRNA	0.0303	0.307	-0.571
CXCL1-mRNA	-0.114	1.17	-2.42
CCND1-mRNA	0.0757	0.787	-1.47
LAMC2-mRNA	0.0832	0.866	-1.62
HK1-mRNA	-0.0271	0.289	-0.593
CCNE1-mRNA	0.0886	0.945	-1.76
HK2-mRNA	-0.0554	0.592	-1.22
POLD1-mRNA	0.0298	0.336	-0.629
SPRY4-mRNA	0.108	1.22	-2.29
TNFSF13-mRNA	0.0365	0.46	-0.865
TLR3-mRNA	0.0658	0.843	-1.59
UBE2T-mRNA	0.0375	0.578	-1.09
PRR5-mRNA	-0.0349	0.581	-1.17
LIF-mRNA	0.0822	1.65	-3.15
CDC25C-mRNA	0.0361	0.949	-1.82
TYMS-mRNA	-0.0203	0.615	-1.23
JAG1-mRNA	-0.0166	0.536	-1.07
BLM-mRNA	-0.0126	0.428	-0.851
DEPTOR-mRNA	0.0166	0.574	-1.11
ENO1-mRNA	-0.0093	0.337	-0.669
SIRPA-mRNA	0.0119	0.601	-1.17
HMGA1-mRNA	0.00465	0.357	-0.695
BCAT1-mRNA	-0.0116	0.979	-1.93
UBE2C-mRNA	-0.00823	0.815	-1.61
RIPK1-mRNA	0.00663	0.679	-1.32
EGR1-mRNA	-0.00373	0.688	-1.35
CD47-mRNA	-0.000645	0.5	-0.98
AQP9-mRNA	-0.000483	0.772	-1.51
PTPN11-mRNA	9.30E-05	0.36	-0.705

Upper confidence limit (log2)	Linear fold change	Lower confidence limit (linear)
7	54.6	23.3
4.73	14.4	7.76
4.5	11.5	5.9
4.62	12.3	6.14
4.61	11.6	5.5
4.99	14.1	6.26
5.5	18.1	7.23
4.79	11.9	5.07
6.61	30.2	9.32
2.94	4.52	2.67
5.69	18.6	6.72
3.75	6.85	3.49
2.98	4.57	2.66
5.4	15.6	5.79
4.44	9.55	4.22
4.61	10.4	4.45
4.21	8.45	3.85
3.47	5.77	3.01
6.15	22.1	6.89
6.13	21.7	6.72
5.99	20.2	6.43
4.79	11.1	4.4
5.25	13.8	5.03
4.26	8.4	3.69
3.61	6.03	2.98
2.14	2.9	1.91
4.9	11.4	4.38
3.76	6.45	3.07
4.74	10.5	4.11
5.83	17.9	5.65
4.81	10.8	4.17
3.6	5.92	2.9
5.17	12.9	4.6
3.61	5.95	2.9
4.11	7.56	3.31
4.61	9.65	3.81
3.76	6.34	2.97
5.33	13.6	4.6
3.32	5.08	2.59
6.22	21	5.9
5.32	13.5	4.54
3.03	4.39	2.36
3.15	4.65	2.44
3.84	6.49	2.95
5.04	11.6	4.14
4.03	7.12	3.11

5.92	17.9	5.28
6.19	20.4	5.7
6.26	21	5.72
3.84	6.45	2.91
4.34	8.2	3.32
-0.858	0.352	0.225
4.34	8.15	3.28
3.04	4.33	2.28
-0.847	0.351	0.222
4.29	7.9	3.19
6.21	19.8	5.29
3.6	5.65	2.63
2.73	3.71	2.08
2.97	4.17	2.21
3.16	4.55	2.32
3.09	4.41	2.28
4.59	8.98	3.36
3.41	5.06	2.41
3.34	4.88	2.36
3.52	5.31	2.45
3.46	5.15	2.41
3.31	4.79	2.31
3.28	4.7	2.28
3.65	5.6	2.5
4.88	9.95	3.36
3.65	5.57	2.47
1.77	2.3	1.54
3.79	5.88	2.5
2.95	3.97	2.03
5.63	13.9	3.87
3.54	5.22	2.33
4.32	7.48	2.8
3.38	4.8	2.22
-0.764	0.352	0.211
3.68	5.51	2.37
2.82	3.68	1.93
3.35	4.72	2.18
3.87	5.97	2.43
3.67	5.43	2.31
5.01	9.99	3.11
2.87	3.74	1.91
5.06	10.2	3.14
5.5	12.5	3.45
3.22	4.39	2.06
5.39	11.8	3.34
5.15	10.5	3.08
3.68	5.35	2.23

2.56	3.2	1.74
3.78	5.57	2.26
3.88	5.84	2.31
3.17	4.22	1.98
3.49	4.86	2.11
4.07	6.32	2.38
2.63	3.29	1.75
3.67	5.25	2.17
4.71	8.42	2.7
4.37	7.19	2.51
-0.46	0.504	0.35
3.48	4.82	2.08
3.38	4.58	2.02
5.44	11.6	3.08
3.5	4.83	2.06
5.01	9.4	2.75
3.93	5.8	2.21
3.82	5.53	2.17
-0.523	0.446	0.286
3.57	4.92	2.03
4.11	6.24	2.25
4.16	6.35	2.26
2.85	3.55	1.74
2.35	2.83	1.57
4.6	7.64	2.41
2.37	2.85	1.57
3.04	3.81	1.77
3.51	4.69	1.94
3.54	4.75	1.94
3.34	4.36	1.87
3.08	3.88	1.78
5.18	9.79	2.64
-0.476	0.457	0.291
5.25	9.89	2.57
2.88	3.51	1.68
2.43	2.88	1.54
5.86	12.8	2.81
3.39	4.36	1.81
4.37	6.68	2.16
3.44	4.42	1.81
4.6	7.31	2.2
4.04	5.72	1.99
3.22	4.02	1.73
2.88	3.46	1.63
4.05	5.69	1.96
2.73	3.23	1.57
5.09	8.87	2.31

4.32	6.34	2.01
3.9	5.29	1.88
5.1	8.82	2.28
4.33	6.35	2
2.83	3.34	1.56
3.95	5.35	1.85
2.88	3.39	1.56
3.8	5	1.79
-0.661	0.28	0.124
3.12	3.72	1.6
5.17	8.85	2.18
-0.487	0.388	0.211
3.66	4.66	1.72
3.64	4.62	1.7
5.67	10.8	2.28
3.39	4.15	1.64
3.39	4.14	1.64
3.28	3.96	1.61
3.7	4.71	1.71
3.14	3.73	1.57
3.11	3.67	1.56
3.74	4.76	1.7
2.68	3.04	1.44
2.45	2.74	1.38
4.86	7.33	1.85
2.96	3.34	1.43
-0.38	0.409	0.218
3.62	4.34	1.53
1.47	1.81	1.19
3.77	4.59	1.54
-0.377	0.39	0.197
3	3.32	1.38
5.51	9.03	1.78
3.17	3.54	1.39
1.39	1.74	1.15
1.22	1.63	1.13
3.26	3.66	1.39
-0.259	0.494	0.292
2.86	3.11	1.33
3.09	3.4	1.36
4.31	5.5	1.52
-0.474	0.265	0.0973
-0.168	0.622	0.435
1.73	1.98	1.18
2.72	2.93	1.3
3.89	4.62	1.44
2.02	2.21	1.21

2.25	2.42	1.23
3.71	4.3	1.41
4.97	7.04	1.58
3.94	4.69	1.43
2.35	2.51	1.23
3.14	3.4	1.31
-0.344	0.328	0.137
2.62	2.75	1.23
3.32	3.61	1.3
4.03	4.71	1.36
-0.202	0.493	0.28
-0.0772	0.764	0.615
-0.219	0.456	0.242
3.48	3.79	1.29
4.04	4.66	1.32
4.08	4.73	1.32
3.97	4.51	1.3
1.75	1.94	1.12
-0.0977	0.679	0.493
-0.146	0.56	0.347
-0.0799	0.725	0.556
1.47	1.75	1.1
3.78	4.18	1.27
1.84	2	1.12
-0.079	0.71	0.532
2.53	2.59	1.16
2.32	2.39	1.15
3.25	3.39	1.2
-0.192	0.415	0.197
-0.117	0.573	0.355
2.75	2.79	1.16
-0.228	0.326	0.125
3.61	3.83	1.2
-0.204	0.345	0.137
3.53	3.68	1.17
2.21	2.26	1.1
2.38	2.4	1.11
2.05	2.12	1.09
1.76	1.91	1.07
-0.0486	0.73	0.55
-0.172	0.317	0.114
2.84	2.82	1.11
2.18	2.21	1.08
3.75	3.83	1.09
4.78	5.52	1.11
2.19	2.18	1.04
2.56	2.49	1.05

2.79	2.69	1.04
3.43	3.37	1.05
-0.0884	0.244	0.0633
-0.0333	0.574	0.337
-0.0766	0.252	0.067
1.8	1.88	1.02
3.22	3.11	1.04
1.59	1.75	1.02
2.8	2.67	1.03
-0.0164	0.646	0.422
-0.0185	0.566	0.324
2.7	2.57	1.02
1.78	1.86	1.01
-0.00272	0.699	0.489
2.04	2.03	1
2.57	2.43	1
0.00145	0.665	0.442
2.21	2.15	0.994
0.015	0.339	0.114
0.02	0.256	0.0648
2.78	2.61	0.99
1.62	1.75	0.992
4.21	4.2	0.954
2.55	2.39	0.969
0.0224	0.672	0.444
3.09	2.86	0.959
0.0584	0.432	0.179
2.31	2.19	0.96
7.03	10.6	0.858
1.52	1.66	0.964
3.95	3.73	0.897
3.33	3.02	0.905
1.62	1.71	0.952
3.41	3.09	0.9
1.48	1.63	0.955
0.0422	0.742	0.534
4.05	3.8	0.874
7.36	11.2	0.765
0.0591	0.7	0.471
0.161	0.398	0.142
1.68	1.73	0.935
0.101	0.569	0.302
0.137	0.502	0.229
1.75	1.76	0.924
1.99	1.9	0.912
1.07	1.41	0.95
0.2	0.388	0.131

0.133	0.534	0.26
3.46	3.04	0.84
0.0934	0.662	0.411
2.62	2.3	0.859
0.202	0.469	0.192
2.17	1.99	0.879
2.17	1.98	0.873
2.49	2.18	0.847
0.285	0.401	0.132
2.65	2.28	0.826
0.91	1.32	0.931
0.178	0.621	0.341
3.06	2.55	0.782
2.03	1.86	0.844
0.152	0.688	0.426
0.382	0.396	0.12
2.83	2.35	0.776
0.117	0.765	0.54
2.71	2.25	0.776
0.308	0.509	0.21
0.504	0.34	0.0817
0.393	0.432	0.142
1.5	1.56	0.865
0.254	0.585	0.287
0.36	0.475	0.176
0.123	0.785	0.567
0.201	0.674	0.396
0.203	0.676	0.398
0.325	0.535	0.229
0.476	0.403	0.117
1.58	1.58	0.84
0.169	0.736	0.482
2.3	1.95	0.775
1.36	1.49	0.859
1.61	1.59	0.83
1.17	1.4	0.872
0.103	0.84	0.656
0.209	0.703	0.427
0.54	0.435	0.13
0.28	0.65	0.348
0.18	0.765	0.517
0.123	0.834	0.639
0.933	1.3	0.883
2.08	1.79	0.755
0.783	0.335	0.0651
2.67	2.09	0.688
2.03	1.76	0.752

0.392	0.591	0.266
0.182	0.785	0.544
0.35	0.63	0.312
2.24	1.84	0.713
0.339	0.657	0.341
2.02	1.73	0.736
1.73	1.6	0.769
0.26	0.727	0.441
1.47	1.49	0.794
0.627	0.488	0.154
2.31	1.84	0.683
2.57	1.96	0.65
2.25	1.8	0.681
2.31	1.83	0.67
0.792	0.439	0.111
1.59	1.51	0.757
2.07	1.71	0.694
0.707	0.492	0.148
1.54	1.48	0.759
0.211	0.811	0.568
1.48	1.46	0.766
0.864	1.25	0.854
0.283	0.762	0.477
0.695	0.517	0.165
0.38	0.698	0.375
2.15	1.73	0.67
0.991	1.29	0.831
0.819	1.23	0.858
0.506	0.629	0.279
0.312	0.77	0.478
1.31	1.38	0.766
2.81	1.99	0.563
1.03	0.431	0.0912
2.51	1.84	0.595
2.47	1.82	0.598
1.8	1.55	0.686
0.602	0.617	0.251
0.963	0.479	0.118
2.32	1.74	0.605
2.7	1.9	0.556
1.29	1.36	0.754
0.627	0.625	0.253
0.835	0.54	0.163
1.11	1.3	0.782
0.545	0.684	0.321
0.841	0.557	0.173
1.17	1.31	0.762

2.62	1.82	0.536
3.14	2.04	0.474
1.65	1.46	0.674
1.77	1.49	0.649
1.15	1.29	0.753
1.51	1.4	0.69
2.02	1.57	0.608
2.16	1.61	0.583
0.365	0.8	0.496
0.422	0.775	0.448
0.351	0.813	0.519
0.654	0.689	0.302
0.934	0.591	0.183
0.878	1.21	0.791
0.598	0.719	0.341
0.608	0.718	0.338
0.62	1.14	0.846
1.5	1.36	0.659
1.58	1.38	0.64
0.893	0.64	0.22
1.37	1.32	0.676
0.751	1.16	0.806
2.25	1.57	0.521
0.816	0.676	0.26
1.64	1.39	0.62
1.95	1.48	0.565
1.54	1.36	0.637
1.93	1.47	0.565
0.455	0.811	0.48
1.65	1.39	0.611
0.888	0.666	0.24
0.316	0.866	0.603
0.511	0.794	0.442
0.817	0.692	0.272
1.01	0.638	0.202
1.35	1.3	0.661
0.882	0.688	0.257
0.422	0.842	0.529
1.16	0.629	0.177
0.411	0.851	0.544
2.34	1.53	0.459
0.53	0.822	0.468
0.966	1.19	0.723
0.82	0.743	0.313
0.28	0.904	0.673
0.718	0.773	0.363
1.02	1.19	0.701

0.885	0.741	0.297
0.727	0.787	0.374
1.59	0.592	0.116
0.339	0.896	0.635
1.67	1.32	0.548
0.977	0.732	0.272
1.61	1.3	0.559
0.67	0.812	0.414
0.338	0.904	0.647
1.45	0.648	0.153
1.96	1.37	0.481
1.55	1.28	0.559
0.786	0.796	0.367
1.43	0.666	0.165
1.22	0.714	0.219
0.336	0.912	0.658
0.512	0.873	0.534
2.12	1.37	0.434
0.865	1.14	0.709
0.595	0.861	0.49
1.45	1.23	0.559
3	0.473	0.0279
0.692	1.1	0.755
1.2	0.752	0.246
0.761	0.838	0.414
2.46	0.568	0.0587
1.01	0.794	0.314
1.43	1.22	0.549
1.86	1.29	0.458
1.31	0.744	0.223
0.599	0.876	0.506
0.571	0.881	0.523
1.05	1.15	0.641
0.677	0.866	0.469
1.26	1.18	0.581
0.777	0.85	0.421
1.04	0.807	0.317
0.479	0.906	0.589
1.31	0.763	0.235
1.02	0.818	0.33
0.85	1.11	0.685
0.613	0.889	0.517
1.21	0.794	0.272
0.637	0.886	0.505
1.11	0.811	0.305
0.997	0.83	0.345
1.12	0.818	0.308

1.3	0.796	0.256
1.93	1.25	0.411
0.601	0.901	0.535
0.869	0.862	0.407
1.39	0.794	0.24
1.18	0.831	0.306
0.571	0.914	0.562
1.44	0.797	0.234
1.37	0.808	0.252
1.21	0.828	0.295
2.89	0.64	0.0553
0.737	1.08	0.702
1.43	1.16	0.501
0.58	0.917	0.563
1.59	1.18	0.461
1.95	0.756	0.147
1.27	0.834	0.288
0.549	0.925	0.585
1.6	0.798	0.21
2.63	1.28	0.266
7.43	2.01	0.0234
1.21	1.12	0.541
1.92	1.19	0.377
1.81	1.18	0.397
0.503	1.05	0.773
0.568	0.937	0.593
1.15	1.1	0.548
1.05	1.09	0.571
0.804	0.921	0.486
5.73	1.55	0.0454
0.557	0.947	0.61
0.642	0.94	0.566
1.93	0.829	0.181
1.33	0.88	0.308
0.49	0.957	0.651
1.06	0.909	0.397
1.91	1.15	0.349
1.35	1.1	0.474
1.64	0.864	0.239
0.867	0.933	0.477
0.55	0.959	0.628
1.97	0.862	0.19
1.08	1.07	0.539
2.24	0.853	0.155
1.02	0.936	0.433
1.43	0.912	0.309
1.85	1.1	0.339

1.29	1.07	0.468
0.425	0.976	0.709
1.42	0.921	0.316
1.81	1.09	0.337
0.914	0.955	0.484
2.1	1.1	0.28
1	0.96	0.46
0.632	1.02	0.673
2.19	0.924	0.187
1.62	1.05	0.362
1.78	1.06	0.326
0.539	0.981	0.663
1.94	1.06	0.294
1.1	0.962	0.431
0.688	1.02	0.647
2.51	1.08	0.204
0.938	1.03	0.549
1.72	1.05	0.333
1.17	1.03	0.468
1.1	0.976	0.444
3.32	1.06	0.112
1.9	1.03	0.282
1.18	0.986	0.428
1.03	0.989	0.477
0.826	0.991	0.554
1.14	1.01	0.464
0.651	0.994	0.629
1.19	1.01	0.446
0.705	1	0.618
1.91	0.992	0.262
1.59	0.994	0.329
1.34	1	0.399
1.35	0.997	0.392
0.979	1	0.507
1.51	1	0.35
0.705	1	0.614

Upper confidence limit (linear)	BY.p.value	-log10(BY.p.value)	method
128	0.0264	1.578396073	lm.nb
26.6	0.0264	1.578396073	lm.nb
22.6	0.055	1.259637311	lm.nb
24.6	0.055	1.259637311	lm.nb
24.4	0.0811	1.090979146	lm.nb
31.8	0.0811	1.090979146	lm.nb
45.4	0.0881	1.055024092	lm.nb
27.7	0.097	1.013228266	lm.nb
97.7	0.097	1.013228266	lm.nb
7.66	0.097	1.013228266	lm.nb
51.7	0.097	1.013228266	lm.nb
13.4	0.097	1.013228266	lm.nb
7.88	0.097	1.013228266	lm.nb
42.2	0.097	1.013228266	lm.nb
21.6	0.097	1.013228266	lm.nb
24.5	0.097	1.013228266	lm.nb
18.5	0.097	1.013228266	lm.nb
11.1	0.097	1.013228266	lm.nb
70.9	0.097	1.013228266	lm.nb
69.9	0.097	1.013228266	lm.nb
63.5	0.097	1.013228266	lm.nb
27.7	0.097	1.013228266	lm.nb
38	0.097	1.013228266	lm.nb
19.1	0.097	1.013228266	lm.nb
12.2	0.097	1.013228266	lm.nb
4.41	0.097	1.013228266	lm.nb
29.8	0.097	1.013228266	lm.nb
13.6	0.097	1.013228266	lm.nb
26.8	0.097	1.013228266	lm.nb
56.7	0.097	1.013228266	lm.nb
28.1	0.097	1.013228266	lm.nb
12.1	0.097	1.013228266	lm.nb
35.9	0.097	1.013228266	lm.nb
12.2	0.097	1.013228266	lm.nb
17.2	0.097	1.013228266	lm.nb
24.4	0.097	1.013228266	lm.nb
13.5	0.097	1.013228266	lm.nb
40.2	0.097	1.013228266	lm.nb
9.97	0.097	1.013228266	lm.nb
74.7	0.097	1.013228266	lm.nb
39.9	0.097	1.013228266	lm.nb
8.16	0.097	1.013228266	lm.nb
8.87	0.097	1.013228266	lm.nb
14.3	0.097	1.013228266	lm.nb
32.8	0.097	1.013228266	lm.nb
16.3	0.097	1.013228266	lm.nb

60.4	0.097	1.013228266	lm.nb
73	0.097	1.013228266	lm.nb
76.8	0.1	1	lm.nb
14.3	0.1	1	lm.nb
20.3	0.101	0.995678626	lm.nb
0.552	0.101	0.995678626	lm.nb
20.3	0.104	0.982966661	lm.nb
8.22	0.104	0.982966661	lm.nb
0.556	0.104	0.982966661	lm.nb
19.6	0.104	0.982966661	lm.nb
73.9	0.104	0.982966661	lm.nb
12.2	0.104	0.982966661	lm.nb
6.63	0.104	0.982966661	lm.nb
7.85	0.104	0.982966661	lm.nb
8.91	0.104	0.982966661	lm.nb
8.54	0.104	0.982966661	lm.nb
24	0.108	0.966576245	lm.nb
10.7	0.121	0.91721463	lm.nb
10.1	0.121	0.91721463	lm.nb
11.5	0.125	0.903089987	lm.nb
11	0.125	0.903089987	lm.nb
9.94	0.127	0.896196279	lm.nb
9.69	0.129	0.88941029	lm.nb
12.6	0.129	0.88941029	lm.nb
29.5	0.133	0.876148359	lm.nb
12.6	0.133	0.876148359	lm.nb
3.42	0.143	0.844663963	lm.nb
13.8	0.147	0.832682665	lm.nb
7.75	0.147	0.832682665	lm.nb
49.7	0.147	0.832682665	lm.nb
11.7	0.149	0.826813732	lm.nb
20	0.149	0.826813732	lm.nb
10.4	0.152	0.818156412	lm.nb
0.589	0.152	0.818156412	lm.nb
12.9	0.155	0.809668302	lm.nb
7.04	0.155	0.809668302	lm.nb
10.2	0.155	0.809668302	lm.nb
14.6	0.163	0.787812396	lm.nb
12.8	0.167	0.777283529	lm.nb
32.1	0.167	0.777283529	lm.nb
7.32	0.167	0.777283529	lm.nb
33.4	0.167	0.777283529	lm.nb
45.3	0.168	0.774690718	lm.nb
9.35	0.17	0.769551079	lm.nb
42	0.17	0.769551079	lm.nb
35.5	0.184	0.735182177	lm.nb
12.8	0.184	0.735182177	lm.nb

5.9	0.187	0.728158393	lm.nb
13.7	0.187	0.728158393	lm.nb
14.8	0.187	0.728158393	lm.nb
9.02	0.187	0.728158393	lm.nb
11.2	0.187	0.728158393	lm.nb
16.8	0.189	0.723538196	lm.nb
6.18	0.189	0.723538196	lm.nb
12.7	0.189	0.723538196	lm.nb
26.2	0.189	0.723538196	lm.nb
20.6	0.189	0.723538196	lm.nb
0.727	0.189	0.723538196	lm.nb
11.2	0.189	0.723538196	lm.nb
10.4	0.192	0.716698771	lm.nb
43.3	0.196	0.707743929	lm.nb
11.3	0.197	0.705533774	lm.nb
32.1	0.204	0.690369833	lm.nb
15.2	0.204	0.690369833	lm.nb
14.1	0.204	0.690369833	lm.nb
0.696	0.209	0.679853714	lm.nb
11.9	0.215	0.66756154	lm.nb
17.3	0.218	0.661543506	lm.nb
17.8	0.22	0.657577319	lm.nb
7.23	0.224	0.649751982	lm.nb
5.1	0.232	0.634512015	lm.nb
24.3	0.233	0.632644079	lm.nb
5.16	0.233	0.632644079	lm.nb
8.21	0.236	0.627087997	lm.nb
11.4	0.236	0.627087997	lm.nb
11.6	0.236	0.627087997	lm.nb
10.1	0.236	0.627087997	lm.nb
8.48	0.236	0.627087997	lm.nb
36.3	0.236	0.627087997	lm.nb
0.719	0.242	0.616184634	lm.nb
38.1	0.263	0.580044252	lm.nb
7.35	0.263	0.580044252	lm.nb
5.38	0.267	0.573488739	lm.nb
58.3	0.269	0.57024772	lm.nb
10.5	0.269	0.57024772	lm.nb
20.7	0.269	0.57024772	lm.nb
10.8	0.283	0.548213564	lm.nb
24.3	0.286	0.543633967	lm.nb
16.4	0.286	0.543633967	lm.nb
9.34	0.286	0.543633967	lm.nb
7.35	0.289	0.539102157	lm.nb
16.5	0.3	0.522878745	lm.nb
6.62	0.301	0.521433504	lm.nb
34	0.303	0.518557371	lm.nb

20	0.312	0.505845406	lm.nb
14.9	0.312	0.505845406	lm.nb
34.2	0.312	0.505845406	lm.nb
20.2	0.318	0.49757288	lm.nb
7.13	0.325	0.488116639	lm.nb
15.5	0.337	0.472370099	lm.nb
7.37	0.341	0.467245621	lm.nb
14	0.343	0.46470588	lm.nb
0.632	0.344	0.463441557	lm.nb
8.67	0.348	0.458420756	lm.nb
35.9	0.348	0.458420756	lm.nb
0.714	0.348	0.458420756	lm.nb
12.7	0.359	0.444905551	lm.nb
12.5	0.362	0.441291429	lm.nb
51	0.362	0.441291429	lm.nb
10.5	0.362	0.441291429	lm.nb
10.5	0.362	0.441291429	lm.nb
9.73	0.362	0.441291429	lm.nb
13	0.362	0.441291429	lm.nb
8.83	0.362	0.441291429	lm.nb
8.65	0.37	0.431798276	lm.nb
13.3	0.37	0.431798276	lm.nb
6.4	0.398	0.400116928	lm.nb
5.48	0.432	0.364516253	lm.nb
29.1	0.455	0.341988603	lm.nb
7.8	0.48	0.318758763	lm.nb
0.768	0.489	0.310691141	lm.nb
12.3	0.498	0.302770657	lm.nb
2.76	0.498	0.302770657	lm.nb
13.7	0.516	0.287350298	lm.nb
0.77	0.532	0.274088368	lm.nb
8.01	0.562	0.250263684	lm.nb
45.7	0.573	0.241845378	lm.nb
9.01	0.576	0.239577517	lm.nb
2.63	0.58	0.236572006	lm.nb
2.33	0.584	0.233587153	lm.nb
9.59	0.584	0.233587153	lm.nb
0.836	0.585	0.232844134	lm.nb
7.27	0.585	0.232844134	lm.nb
8.5	0.591	0.228412519	lm.nb
19.8	0.593	0.226945307	lm.nb
0.72	0.593	0.226945307	lm.nb
0.89	0.593	0.226945307	lm.nb
3.32	0.593	0.226945307	lm.nb
6.58	0.593	0.226945307	lm.nb
14.8	0.609	0.215382707	lm.nb
4.05	0.61	0.214670165	lm.nb

4.76	0.613	0.212539525	lm.nb
13.1	0.615	0.211124884	lm.nb
31.4	0.615	0.211124884	lm.nb
15.3	0.616	0.210419288	lm.nb
5.1	0.631	0.199970641	lm.nb
8.82	0.649	0.187755303	lm.nb
0.788	0.672	0.172630727	lm.nb
6.15	0.697	0.156767222	lm.nb
10	0.697	0.156767222	lm.nb
16.3	0.708	0.149966742	lm.nb
0.869	0.708	0.149966742	lm.nb
0.948	0.708	0.149966742	lm.nb
0.859	0.722	0.141462802	lm.nb
11.2	0.737	0.132532512	lm.nb
16.4	0.762	0.118045029	lm.nb
17	0.762	0.118045029	lm.nb
15.6	0.765	0.116338565	lm.nb
3.36	0.765	0.116338565	lm.nb
0.935	0.765	0.116338565	lm.nb
0.904	0.765	0.116338565	lm.nb
0.946	0.768	0.11463878	lm.nb
2.77	0.769	0.11407366	lm.nb
13.7	0.773	0.111820506	lm.nb
3.57	0.8	0.096910013	lm.nb
0.947	0.8	0.096910013	lm.nb
5.78	0.804	0.094743951	lm.nb
4.98	0.804	0.094743951	lm.nb
9.52	0.814	0.089375595	lm.nb
0.875	0.815	0.088842391	lm.nb
0.922	0.834	0.078833949	lm.nb
6.74	0.842	0.074687909	lm.nb
0.854	0.842	0.074687909	lm.nb
12.2	0.852	0.069560405	lm.nb
0.868	0.863	0.063989204	lm.nb
11.5	0.9	0.045757491	lm.nb
4.63	0.913	0.039529222	lm.nb
5.21	0.913	0.039529222	lm.nb
4.14	0.917	0.037630664	lm.nb
3.38	0.929	0.031984286	lm.nb
0.967	0.936	0.028724151	lm.nb
0.887	0.942	0.025949097	lm.nb
7.16	0.946	0.024108864	lm.nb
4.54	0.964	0.015922966	lm.nb
13.5	1	0	lm.nb
27.6	1	0	lm.nb
4.56	1	0	lm.nb
5.89	1	0	lm.nb

6.91	1	0	lm.nb
10.8	1	0	lm.nb
0.941	1	0	lm.nb
0.977	1	0	lm.nb
0.948	1	0	lm.nb
3.47	1	0	lm.nb
9.35	1	0	lm.nb
3.01	1	0	lm.nb
6.94	1	0	lm.nb
0.989	1	0	lm.nb
0.987	1	0	lm.nb
6.48	1	0	lm.nb
3.44	1	0	lm.nb
0.998	1	0	lm.nb
4.12	1	0	lm.nb
5.92	1	0	lm.nb
1	1	0	lm.nb
4.63	1	0	lm.nb
1.01	1	0	lm.nb
1.01	1	0	lm.nb
6.89	1	0	lm.nb
3.08	1	0	lm.nb
18.5	1	0	lm.nb
5.88	1	0	lm.nb
1.02	1	0	lm.nb
8.5	1	0	lm.nb
1.04	1	0	lm.nb
4.97	1	0	lm.nb
131	1	0	loglinear
2.87	1	0	lm.nb
15.5	1	0	lm.nb
10.1	1	0	lm.nb
3.08	1	0	lm.nb
10.6	1	0	lm.nb
2.78	1	0	lm.nb
1.03	1	0	lm.nb
16.5	1	0	lm.nb
165	1	0	loglinear
1.04	1	0	lm.nb
1.12	1	0	lm.nb
3.2	1	0	lm.nb
1.07	1	0	lm.nb
1.1	1	0	lm.nb
3.36	1	0	lm.nb
3.97	1	0	lm.nb
2.1	1	0	lm.nb
1.15	1	0	lm.nb

1.1	1	0	lm.nb
11	1	0	lm.nb
1.07	1	0	lm.nb
6.15	1	0	lm.nb
1.15	1	0	lm.nb
4.5	1	0	lm.nb
4.49	1	0	lm.nb
5.61	1	0	lm.nb
1.22	1	0	lm.nb
6.27	1	0	lm.nb
1.88	1	0	lm.nb
1.13	1	0	lm.nb
8.31	1	0	lm.nb
4.08	1	0	lm.nb
1.11	1	0	lm.nb
1.3	1	0	lm.nb
7.09	1	0	lm.nb
1.08	1	0	lm.nb
6.53	1	0	lm.nb
1.24	1	0	lm.nb
1.42	1	0	lm.nb
1.31	1	0	lm.nb
2.83	1	0	lm.nb
1.19	1	0	lm.nb
1.28	1	0	lm.nb
1.09	1	0	lm.nb
1.15	1	0	lm.nb
1.15	1	0	lm.nb
1.25	1	0	lm.nb
1.39	1	0	lm.nb
2.99	1	0	lm.nb
1.12	1	0	lm.nb
4.92	1	0	lm.nb
2.57	1	0	lm.nb
3.05	1	0	lm.nb
2.24	1	0	lm.nb
1.07	1	0	lm.nb
1.16	1	0	lm.nb
1.45	1	0	lm.nb
1.21	1	0	lm.nb
1.13	1	0	lm.nb
1.09	1	0	lm.nb
1.91	1	0	lm.nb
4.23	1	0	lm.nb
1.72	1	0	lm.nb
6.37	1	0	lm.nb
4.1	1	0	lm.nb

1.31	1	0	lm.nb
1.13	1	0	lm.nb
1.27	1	0	lm.nb
4.74	1	0	lm.nb
1.26	1	0	lm.nb
4.04	1	0	lm.nb
3.31	1	0	lm.nb
1.2	1	0	lm.nb
2.78	1	0	lm.nb
1.54	1	0	lm.nb
4.95	1	0	lm.nb
5.94	1	0	lm.nb
4.75	1	0	lm.nb
4.98	1	0	lm.nb
1.73	1	0	lm.nb
3.01	1	0	lm.nb
4.21	1	0	lm.nb
1.63	1	0	lm.nb
2.9	1	0	lm.nb
1.16	1	0	lm.nb
2.79	1	0	lm.nb
1.82	1	0	lm.nb
1.22	1	0	lm.nb
1.62	1	0	lm.nb
1.3	1	0	lm.nb
4.44	1	0	lm.nb
1.99	1	0	lm.nb
1.76	1	0	lm.nb
1.42	1	0	lm.nb
1.24	1	0	lm.nb
2.48	1	0	lm.nb
7.02	1	0	lm.nb
2.04	1	0	lm.nb
5.69	1	0	lm.nb
5.54	1	0	lm.nb
3.49	1	0	lm.nb
1.52	1	0	lm.nb
1.95	1	0	lm.nb
5	1	0	lm.nb
6.49	1	0	lm.nb
2.45	1	0	lm.nb
1.54	1	0	lm.nb
1.78	1	0	lm.nb
2.15	1	0	lm.nb
1.46	1	0	lm.nb
1.79	1	0	lm.nb
2.25	1	0	lm.nb

6.17	1	0	lm.nb
8.79	1	0	lm.nb
3.14	1	0	lm.nb
3.4	1	0	lm.nb
2.23	1	0	lm.nb
2.84	1	0	lm.nb
4.06	1	0	lm.nb
4.46	1	0	lm.nb
1.29	1	0	lm.nb
1.34	1	0	lm.nb
1.28	1	0	lm.nb
1.57	1	0	lm.nb
1.91	1	0	lm.nb
1.84	1	0	lm.nb
1.51	1	0	lm.nb
1.52	1	0	lm.nb
1.54	1	0	lm.nb
2.82	1	0	lm.nb
2.99	1	0	lm.nb
1.86	1	0	lm.nb
2.58	1	0	lm.nb
1.68	1	0	lm.nb
4.76	1	0	lm.nb
1.76	1	0	lm.nb
3.12	1	0	lm.nb
3.87	1	0	lm.nb
2.9	1	0	lm.nb
3.8	1	0	lm.nb
1.37	1	0	lm.nb
3.14	1	0	lm.nb
1.85	1	0	lm.nb
1.24	1	0	lm.nb
1.42	1	0	lm.nb
1.76	1	0	lm.nb
2.02	1	0	lm.nb
2.54	1	0	lm.nb
1.84	1	0	lm.nb
1.34	1	0	lm.nb
2.23	1	0	lm.nb
1.33	1	0	lm.nb
5.08	1	0	lm.nb
1.44	1	0	lm.nb
1.95	1	0	lm.nb
1.77	1	0	lm.nb
1.21	1	0	lm.nb
1.64	1	0	lm.nb
2.02	1	0	lm.nb

1.85	1	0	lm.nb
1.66	1	0	lm.nb
3.02	1	0	lm.nb
1.26	1	0	lm.nb
3.19	1	0	lm.nb
1.97	1	0	lm.nb
3.05	1	0	lm.nb
1.59	1	0	lm.nb
1.26	1	0	lm.nb
2.74	1	0	lm.nb
3.88	1	0	lm.nb
2.93	1	0	lm.nb
1.72	1	0	lm.nb
2.69	1	0	lm.nb
2.32	1	0	lm.nb
1.26	1	0	lm.nb
1.43	1	0	lm.nb
4.35	1	0	lm.nb
1.82	1	0	lm.nb
1.51	1	0	lm.nb
2.72	1	0	lm.nb
8	1	0	loglinear
1.62	1	0	lm.nb
2.3	1	0	lm.nb
1.69	1	0	lm.nb
5.5	1	0	loglinear
2.01	1	0	lm.nb
2.7	1	0	lm.nb
3.63	1	0	lm.nb
2.49	1	0	lm.nb
1.51	1	0	lm.nb
1.49	1	0	lm.nb
2.07	1	0	lm.nb
1.6	1	0	lm.nb
2.39	1	0	lm.nb
1.71	1	0	lm.nb
2.05	1	0	lm.nb
1.39	1	0	lm.nb
2.48	1	0	lm.nb
2.03	1	0	lm.nb
1.8	1	0	lm.nb
1.53	1	0	lm.nb
2.31	1	0	lm.nb
1.55	1	0	lm.nb
2.16	1	0	lm.nb
2	1	0	lm.nb
2.17	1	0	lm.nb

2.47	1	0	lm.nb
3.81	1	0	lm.nb
1.52	1	0	lm.nb
1.83	1	0	lm.nb
2.62	1	0	lm.nb
2.26	1	0	lm.nb
1.49	1	0	lm.nb
2.72	1	0	lm.nb
2.59	1	0	lm.nb
2.32	1	0	lm.nb
7.4	1	0	loglinear
1.67	1	0	lm.nb
2.7	1	0	lm.nb
1.49	1	0	lm.nb
3	1	0	lm.nb
3.87	1	0	lm.nb
2.41	1	0	lm.nb
1.46	1	0	lm.nb
3.03	1	0	lm.nb
6.18	1	0	lm.nb
172	1	0	loglinear
2.31	1	0	lm.nb
3.78	1	0	lm.nb
3.51	1	0	lm.nb
1.42	1	0	lm.nb
1.48	1	0	lm.nb
2.21	1	0	lm.nb
2.07	1	0	lm.nb
1.75	1	0	lm.nb
53.2	1	0	loglinear
1.47	1	0	lm.nb
1.56	1	0	lm.nb
3.81	1	0	lm.nb
2.52	1	0	lm.nb
1.4	1	0	lm.nb
2.08	1	0	lm.nb
3.77	1	0	lm.nb
2.56	1	0	lm.nb
3.12	1	0	lm.nb
1.82	1	0	lm.nb
1.46	1	0	lm.nb
3.92	1	0	lm.nb
2.11	1	0	lm.nb
4.71	1	0	lm.nb
2.03	1	0	lm.nb
2.69	1	0	lm.nb
3.59	1	0	lm.nb

2.45	1	0	lm.nb
1.34	1	0	lm.nb
2.68	1	0	lm.nb
3.51	1	0	lm.nb
1.88	1	0	lm.nb
4.3	1	0	lm.nb
2	1	0	lm.nb
1.55	1	0	lm.nb
4.56	1	0	lm.nb
3.07	1	0	lm.nb
3.44	1	0	lm.nb
1.45	1	0	lm.nb
3.84	1	0	lm.nb
2.15	1	0	lm.nb
1.61	1	0	lm.nb
5.69	1	0	lm.nb
1.92	1	0	lm.nb
3.29	1	0	lm.nb
2.25	1	0	lm.nb
2.15	1	0	lm.nb
9.97	1	0	loglinear
3.72	1	0	lm.nb
2.27	1	0	lm.nb
2.05	1	0	lm.nb
1.77	1	0	lm.nb
2.21	1	0	lm.nb
1.57	1	0	lm.nb
2.28	1	0	lm.nb
1.63	1	0	lm.nb
3.75	1	0	lm.nb
3.01	1	0	lm.nb
2.53	1	0	lm.nb
2.54	1	0	lm.nb
1.97	1	0	lm.nb
2.85	1	0	lm.nb
1.63	1	0	lm.nb

Gene.sets
Cytokine and Chemokine Signaling, Lymphoid Compartment

Antigen Presentation, Immune Cell Adhesion and Migration, Lymphoid Compartment
Cytotoxicity, Lymphoid Compartment
Cytokine and Chemokine Signaling, Myeloid Compartment
Interferon Signaling
Antigen Presentation, Cytotoxicity, Hypoxia, Interferon Signaling, JAK-STAT Signaling, Lymphoid Compartment, TG
Antigen Presentation
Cytokine and Chemokine Signaling, Lymphoid Compartment
Myeloid Compartment
Costimulatory Signaling, Cytokine and Chemokine Signaling, JAK-STAT Signaling, PI3K-Akt
Myeloid Compartment

Cytotoxicity, Lymphoid Compartment

Cytotoxicity, Lymphoid Compartment
Antigen Presentation, Cytokine and Chemokine Signaling

Cytokine and Chemokine Signaling
Antigen Presentation, Cytotoxicity, Lymphoid Compartment

Costimulatory Signaling, Lymphoid Compartment
Costimulatory Signaling, Immune Cell Adhesion and Migration
Apoptosis, Hypoxia, Myeloid Compartment, PI3K-Akt
Interferon Signaling
Apoptosis

Cytokine and Chemokine Signaling, JAK-STAT Signaling
Costimulatory Signaling, Cytokine and Chemokine Signaling, JAK-STAT Signaling, Lymphoid Compartment, PI3K-Ak
Cytotoxicity, Lymphoid Compartment
Lymphoid Compartment
Angiogenesis
Cytokine and Chemokine Signaling, Lymphoid Compartment
Cytokine and Chemokine Signaling
Costimulatory Signaling, Immune Cell Adhesion and Migration, Lymphoid Compartment
Immune Cell Adhesion and Migration, Matrix Remodeling and Metastasis
Cytokine and Chemokine Signaling, Myeloid Compartment
Costimulatory Signaling, Immune Cell Adhesion and Migration, Lymphoid Compartment
Apoptosis, Myeloid Compartment
Cytokine and Chemokine Signaling, Lymphoid Compartment
Cytotoxicity, MAPK, NF-kappaB Signaling, PI3K-Akt
Antigen Presentation, Matrix Remodeling and Metastasis
Cytokine and Chemokine Signaling, Cytotoxicity, Epigenetic Regulation, Interferon Signaling, JAK-STAT Signaling, L
Antigen Presentation, Hypoxia, Immune Cell Adhesion and Migration, Myeloid Compartment
Cytotoxicity, Lymphoid Compartment
Cytokine and Chemokine Signaling, Hypoxia, Immune Cell Adhesion and Migration, JAK-STAT Signaling, MAPK, Me

Costimulatory Signaling, Lymphoid Compartment
Costimulatory Signaling, Lymphoid Compartment
Cytotoxicity, Interferon Signaling, Lymphoid Compartment
Costimulatory Signaling, Immune Cell Adhesion and Migration

Cell Proliferation, DNA Damage Repair
Costimulatory Signaling, Myeloid Compartment
Immune Cell Adhesion and Migration, Matrix Remodeling and Metastasis, Myeloid Compartment
Metabolic Stress

Lymphoid Compartment

Cytokine and Chemokine Signaling, Myeloid Compartment
Cytokine and Chemokine Signaling, Cytotoxicity, Interferon Signaling, JAK-STAT Signaling, Lymphoid Compartment

Myeloid Compartment
Costimulatory Signaling, Lymphoid Compartment

Angiogenesis, Cell Proliferation, JAK-STAT Signaling, PI3K-Akt, Wnt Signaling
Myeloid Compartment
Apoptosis

Immune Cell Adhesion and Migration, Matrix Remodeling and Metastasis
NF-kappaB Signaling
Immune Cell Adhesion and Migration
Myeloid Compartment

Antigen Presentation, Interferon Signaling
Costimulatory Signaling, Immune Cell Adhesion and Migration, Lymphoid Compartment
Antigen Presentation, Interferon Signaling, Myeloid Compartment
Antigen Presentation, Interferon Signaling

Angiogenesis, Matrix Remodeling and Metastasis
Costimulatory Signaling, Immune Cell Adhesion and Migration, Lymphoid Compartment
Metabolic Stress
MAPK, Matrix Remodeling and Metastasis, TGF-beta Signaling

Cytotoxicity, Lymphoid Compartment
Immune Cell Adhesion and Migration, Matrix Remodeling and Metastasis
Costimulatory Signaling, Immune Cell Adhesion and Migration, Lymphoid Compartment

Immune Cell Adhesion and Migration, Matrix Remodeling and Metastasis, PI3K-Akt
Costimulatory Signaling, Immune Cell Adhesion and Migration, Lymphoid Compartment
Costimulatory Signaling, Lymphoid Compartment
Antigen Presentation, Apoptosis, Cell Proliferation, Costimulatory Signaling, Hedgehog Signaling, NF-kappaB Signa

Antigen Presentation, Immune Cell Adhesion and Migration, Interferon Signaling
Antigen Presentation, Costimulatory Signaling, Immune Cell Adhesion and Migration, Interferon Signaling
Cytokine and Chemokine Signaling
Apoptosis, MAPK, Myeloid Compartment
Cytokine and Chemokine Signaling, MAPK, Myeloid Compartment, PI3K-Akt
Cytotoxicity, Interferon Signaling
Myeloid Compartment
Cell Proliferation, Hypoxia, JAK-STAT Signaling, Metabolic Stress, Myeloid Compartment, PI3K-Akt
Lymphoid Compartment
Interferon Signaling
DNA Damage Repair
Antigen Presentation, MAPK, Metabolic Stress, NF-kappaB Signaling, TGF-beta Signaling
Interferon Signaling
Costimulatory Signaling

Costimulatory Signaling, Lymphoid Compartment, NF-kappaB Signaling

Epigenetic Regulation, Notch Signaling
Antigen Presentation
Antigen Presentation, Cytokine and Chemokine Signaling, Myeloid Compartment
Lymphoid Compartment
Antigen Presentation, Immune Cell Adhesion and Migration, Interferon Signaling
Antigen Presentation, Immune Cell Adhesion and Migration, Interferon Signaling
Immune Cell Adhesion and Migration, Interferon Signaling, Matrix Remodeling and Metastasis
Costimulatory Signaling, Interferon Signaling, Matrix Remodeling and Metastasis
Metabolic Stress

Cytokine and Chemokine Signaling, Cytotoxicity, JAK-STAT Signaling, PI3K-Akt

Myeloid Compartment, PI3K-Akt
Costimulatory Signaling, JAK-STAT Signaling

Cytokine and Chemokine Signaling, Lymphoid Compartment

Autophagy, Cytokine and Chemokine Signaling, Hypoxia, Immune Cell Adhesion and Migration, JAK-STAT Signaling
Cytokine and Chemokine Signaling, Myeloid Compartment
Cytokine and Chemokine Signaling, Hypoxia, Immune Cell Adhesion and Migration, JAK-STAT Signaling, MAPK, Me
Costimulatory Signaling
Costimulatory Signaling
Lymphoid Compartment
Cytokine and Chemokine Signaling
Immune Cell Adhesion and Migration, Matrix Remodeling and Metastasis, Myeloid Compartment
Antigen Presentation
Costimulatory Signaling, Lymphoid Compartment

Costimulatory Signaling, Lymphoid Compartment, MAPK

Antigen Presentation, Immune Cell Adhesion and Migration, Interferon Signaling
Costimulatory Signaling, Immune Cell Adhesion and Migration, Lymphoid Compartment

NF-kappaB Signaling

Antigen Presentation, Interferon Signaling, JAK-STAT Signaling
Cytokine and Chemokine Signaling, Cytotoxicity, JAK-STAT Signaling
Antigen Presentation, Costimulatory Signaling, Immune Cell Adhesion and Migration, Interferon Signaling

Metabolic Stress
Antigen Presentation, Immune Cell Adhesion and Migration, Interferon Signaling
Antigen Presentation, Costimulatory Signaling, Immune Cell Adhesion and Migration, Interferon Signaling
Costimulatory Signaling, Lymphoid Compartment
Cytotoxicity, Interferon Signaling

Immune Cell Adhesion and Migration, Myeloid Compartment

Cytotoxicity, Interferon Signaling
Costimulatory Signaling
Cytotoxicity, Interferon Signaling

Cytokine and Chemokine Signaling, JAK-STAT Signaling, PI3K-Akt
Antigen Presentation

Cytotoxicity, DNA Damage Repair, Interferon Signaling, Lymphoid Compartment
MAPK, NF-kappaB Signaling
Cytotoxicity, Interferon Signaling, Lymphoid Compartment
Epigenetic Regulation, MAPK
Antigen Presentation, Immune Cell Adhesion and Migration
Myeloid Compartment
Interferon Signaling
Interferon Signaling
Interferon Signaling
Myeloid Compartment

Apoptosis, Autophagy, Hypoxia, JAK-STAT Signaling, PI3K-Akt
Cytotoxicity, Interferon Signaling
Myeloid Compartment
Costimulatory Signaling, Immune Cell Adhesion and Migration
Epigenetic Regulation
Costimulatory Signaling
Apoptosis, Cytotoxicity
Antigen Presentation, Costimulatory Signaling, Immune Cell Adhesion and Migration
Immune Cell Adhesion and Migration, Lymphoid Compartment, Matrix Remodeling and Metastasis, PI3K-Akt

Matrix Remodeling and Metastasis

Immune Cell Adhesion and Migration
Cytokine and Chemokine Signaling
Notch Signaling
Myeloid Compartment

Apoptosis, Autophagy
Autophagy, Costimulatory Signaling, Hypoxia, JAK-STAT Signaling, Metabolic Stress, PI3K-Akt
Autophagy, Metabolic Stress, PI3K-Akt
NF-kappaB Signaling
Antigen Presentation, Myeloid Compartment
Cytokine and Chemokine Signaling

Costimulatory Signaling, Cytokine and Chemokine Signaling, Hypoxia, MAPK, Metabolic Stress, NF-kappaB Signalin

Apoptosis
PI3K-Akt
Cytokine and Chemokine Signaling, MAPK, Myeloid Compartment
Interferon Signaling
Apoptosis, PI3K-Akt
Cell Proliferation, Metabolic Stress

DNA Damage Repair
Cell Proliferation, Metabolic Stress, PI3K-Akt
TGF-beta Signaling
Angiogenesis, Immune Cell Adhesion and Migration, Matrix Remodeling and Metastasis
Cell Proliferation, Hypoxia, Immune Cell Adhesion and Migration, MAPK, Matrix Remodeling and Metastasis, Meta
Interferon Signaling
Angiogenesis
Cytotoxicity, Interferon Signaling
Costimulatory Signaling, Cytokine and Chemokine Signaling, NF-kappaB Signaling
Cytokine and Chemokine Signaling, MAPK, Myeloid Compartment, PI3K-Akt
Costimulatory Signaling, Wnt Signaling
Epigenetic Regulation
Autophagy, Costimulatory Signaling, MAPK, Wnt Signaling

Cytotoxicity, Interferon Signaling
Metabolic Stress
Hedgehog Signaling, Wnt Signaling
Lymphoid Compartment
Antigen Presentation, Apoptosis, Cell Proliferation, Costimulatory Signaling, Hedgehog Signaling, Interferon Signal

Cytokine and Chemokine Signaling, JAK-STAT Signaling, Myeloid Compartment, PI3K-Akt
Myeloid Compartment
Immune Cell Adhesion and Migration
DNA Damage Repair

Immune Cell Adhesion and Migration, Matrix Remodeling and Metastasis
Myeloid Compartment

Cytotoxicity, Interferon Signaling, Lymphoid Compartment

Autophagy, Costimulatory Signaling, Cytokine and Chemokine Signaling, Hypoxia, Immune Cell Adhesion and Migr
Antigen Presentation, Costimulatory Signaling, Immune Cell Adhesion and Migration, Interferon Signaling
Cell Proliferation, JAK-STAT Signaling, PI3K-Akt, Wnt Signaling
Metabolic Stress

Autophagy, Hypoxia, Metabolic Stress, PI3K-Akt, TGF-beta Signaling

Antigen Presentation, Immune Cell Adhesion and Migration

Cytokine and Chemokine Signaling, Hypoxia, JAK-STAT Signaling, Metabolic Stress, PI3K-Akt
MAPK
Angiogenesis, MAPK, PI3K-Akt
Cytotoxicity, Interferon Signaling
MAPK, Metabolic Stress, PI3K-Akt
Metabolic Stress, PI3K-Akt
Lymphoid Compartment
Costimulatory Signaling, Immune Cell Adhesion and Migration, Lymphoid Compartment

NF-kappaB Signaling

Cytokine and Chemokine Signaling, Myeloid Compartment
Cell Proliferation, Metabolic Stress
Antigen Presentation, Apoptosis, Cell Proliferation, Costimulatory Signaling, Hedgehog Signaling, NF-kappaB Signa
Lymphoid Compartment, PI3K-Akt
Lymphoid Compartment
Apoptosis, Autophagy, MAPK, PI3K-Akt
Autophagy, Metabolic Stress, PI3K-Akt
NF-kappaB Signaling

Notch Signaling
Antigen Presentation
Costimulatory Signaling, Cytokine and Chemokine Signaling, JAK-STAT Signaling

Immune Cell Adhesion and Migration

Angiogenesis
Cell Proliferation, DNA Damage Repair, Metabolic Stress
Immune Cell Adhesion and Migration
Immune Cell Adhesion and Migration, Myeloid Compartment

Autophagy, Cytokine and Chemokine Signaling, Hedgehog Signaling, MAPK, Wnt Signaling
Cytokine and Chemokine Signaling
Antigen Presentation, Apoptosis, Cell Proliferation, Costimulatory Signaling, Hedgehog Signaling, NF-kappaB Signa
Metabolic Stress
Cytotoxicity, Interferon Signaling
Hypoxia, Interferon Signaling, JAK-STAT Signaling
Hypoxia, Metabolic Stress

Cell Proliferation, Metabolic Stress, TGF-beta Signaling
Metabolic Stress
Notch Signaling
Cytotoxicity, Interferon Signaling, Lymphoid Compartment
Autophagy, Cytokine and Chemokine Signaling, MAPK, Metabolic Stress, PI3K-Akt
MAPK
Cell Proliferation
Hedgehog Signaling, Wnt Signaling
Angiogenesis, Hypoxia, MAPK, Metabolic Stress, PI3K-Akt
Antigen Presentation, Notch Signaling
Immune Cell Adhesion and Migration, Lymphoid Compartment
Angiogenesis, MAPK, Metabolic Stress, PI3K-Akt
DNA Damage Repair
Notch Signaling

Myeloid Compartment
Interferon Signaling
Metabolic Stress
Myeloid Compartment
Cytotoxicity, Interferon Signaling, JAK-STAT Signaling, Lymphoid Compartment

MAPK, NF-kappaB Signaling
Epigenetic Regulation
MAPK
Angiogenesis, MAPK, PI3K-Akt
Angiogenesis, Antigen Presentation, Immune Cell Adhesion and Migration, Matrix Remodeling and Metastasis, PI3
Angiogenesis
Angiogenesis
Autophagy, Hypoxia, Metabolic Stress, Notch Signaling

Myeloid Compartment
Costimulatory Signaling, Matrix Remodeling and Metastasis, PI3K-Akt
Cell Proliferation

Epigenetic Regulation, Notch Signaling
Cell Proliferation, DNA Damage Repair, Metabolic Stress

Cytotoxicity, Interferon Signaling, Lymphoid Compartment
Angiogenesis
Apoptosis, Cell Proliferation, DNA Damage Repair, MAPK, Metabolic Stress, Notch Signaling, PI3K-Akt, Wnt Signali
Apoptosis
Interferon Signaling, JAK-STAT Signaling, PI3K-Akt
Epigenetic Regulation, Notch Signaling
Matrix Remodeling and Metastasis
Cytotoxicity, Interferon Signaling
Metabolic Stress
Antigen Presentation, Interferon Signaling
Costimulatory Signaling, Cytokine and Chemokine Signaling
Matrix Remodeling and Metastasis, PI3K-Akt
Antigen Presentation, DNA Damage Repair, Interferon Signaling
Antigen Presentation, Cell Proliferation
Immune Cell Adhesion and Migration, Matrix Remodeling and Metastasis, PI3K-Akt
NF-kappaB Signaling
Apoptosis, Immune Cell Adhesion and Migration, Wnt Signaling

MAPK, TGF-beta Signaling

Immune Cell Adhesion and Migration, Matrix Remodeling and Metastasis, PI3K-Akt
Immune Cell Adhesion and Migration
Cell Proliferation, DNA Damage Repair, Metabolic Stress
Cell Proliferation, DNA Damage Repair, JAK-STAT Signaling
Autophagy, Costimulatory Signaling, Metabolic Stress, PI3K-Akt
Autophagy, Costimulatory Signaling, Cytokine and Chemokine Signaling, Hypoxia, Immune Cell Adhesion and Migr
Costimulatory Signaling, NF-kappaB Signaling
Antigen Presentation, Matrix Remodeling and Metastasis
Hypoxia, MAPK, Metabolic Stress, PI3K-Akt

Autophagy, Epigenetic Regulation
Hypoxia, Metabolic Stress
Immune Cell Adhesion and Migration, Interferon Signaling, Matrix Remodeling and Metastasis
Antigen Presentation, Immune Cell Adhesion and Migration
Cytokine and Chemokine Signaling, MAPK
Cytokine and Chemokine Signaling, Cytotoxicity, Interferon Signaling, JAK-STAT Signaling, Lymphoid Compartment

Matrix Remodeling and Metastasis
Costimulatory Signaling, Cytokine and Chemokine Signaling, MAPK, NF-kappaB Signaling, PI3K-Akt
Interferon Signaling, MAPK
Cytokine and Chemokine Signaling, Lymphoid Compartment
MAPK, TGF-beta Signaling

Antigen Presentation
MAPK, Matrix Remodeling and Metastasis, TGF-beta Signaling
Cytokine and Chemokine Signaling, Immune Cell Adhesion and Migration
Myeloid Compartment
Costimulatory Signaling, MAPK
Costimulatory Signaling, Cytokine and Chemokine Signaling, MAPK, NF-kappaB Signaling, PI3K-Akt
MAPK
Apoptosis
Costimulatory Signaling, Metabolic Stress
MAPK

Cell Proliferation, DNA Damage Repair, PI3K-Akt
Apoptosis, Cytokine and Chemokine Signaling, Immune Cell Adhesion and Migration, TGF-beta Signaling
Apoptosis
Cytotoxicity, Interferon Signaling
Immune Cell Adhesion and Migration, Matrix Remodeling and Metastasis
NF-kappaB Signaling
Cell Proliferation
Myeloid Compartment

Autophagy, Cytokine and Chemokine Signaling, MAPK, Metabolic Stress, PI3K-Akt

Apoptosis
Wnt Signaling
Angiogenesis, Immune Cell Adhesion and Migration, Matrix Remodeling and Metastasis
Cell Proliferation

Cytotoxicity, Interferon Signaling, Lymphoid Compartment
DNA Damage Repair
DNA Damage Repair

Epigenetic Regulation, Notch Signaling
Angiogenesis, Cell Proliferation, Metabolic Stress, Notch Signaling
DNA Damage Repair
Costimulatory Signaling
Costimulatory Signaling, Lymphoid Compartment
Cell Proliferation, PI3K-Akt, TGF-beta Signaling
Lymphoid Compartment
MAPK, NF-kappaB Signaling
Apoptosis, Cytotoxicity
Costimulatory Signaling, Immune Cell Adhesion and Migration
Cytokine and Chemokine Signaling, Hypoxia, JAK-STAT Signaling, PI3K-Akt
DNA Damage Repair

Metabolic Stress, PI3K-Akt

Apoptosis
Immune Cell Adhesion and Migration
Cell Proliferation, DNA Damage Repair, Metabolic Stress

Hedgehog Signaling, Wnt Signaling
Epigenetic Regulation
Epigenetic Regulation, Notch Signaling

Cell Proliferation, DNA Damage Repair
DNA Damage Repair
Metabolic Stress
Matrix Remodeling and Metastasis, TGF-beta Signaling
Wnt Signaling

Wnt Signaling
Cytotoxicity, Interferon Signaling, JAK-STAT Signaling, Lymphoid Compartment, PI3K-Akt
Hypoxia, Metabolic Stress, PI3K-Akt
Costimulatory Signaling, Cytokine and Chemokine Signaling, MAPK, Metabolic Stress, NF-kappaB Signaling, PI3K-A
Angiogenesis
Apoptosis, Autophagy, Costimulatory Signaling, Cytokine and Chemokine Signaling, Hypoxia, JAK-STAT Signaling, M
Immune Cell Adhesion and Migration, Interferon Signaling, Matrix Remodeling and Metastasis
Notch Signaling
Matrix Remodeling and Metastasis, Wnt Signaling
Notch Signaling

Cytokine and Chemokine Signaling, Metabolic Stress
Cell Proliferation, DNA Damage Repair
Metabolic Stress
Metabolic Stress

Costimulatory Signaling, Cytokine and Chemokine Signaling, Hypoxia, MAPK, Metabolic Stress, NF-kappaB Signalin
Autophagy, Cytokine and Chemokine Signaling, JAK-STAT Signaling, MAPK, Metabolic Stress, PI3K-Akt
Cell Proliferation, DNA Damage Repair
DNA Damage Repair
MAPK
Notch Signaling
Apoptosis, MAPK, Matrix Remodeling and Metastasis
Hypoxia
Matrix Remodeling and Metastasis, Myeloid Compartment, PI3K-Akt
Cell Proliferation
Hypoxia, Metabolic Stress
Apoptosis, Immune Cell Adhesion and Migration, Matrix Remodeling and Metastasis

Matrix Remodeling and Metastasis, PI3K-Akt
Antigen Presentation, Cell Proliferation
Metabolic Stress, Myeloid Compartment
Apoptosis, Cell Proliferation

Cell Proliferation
Cell Proliferation, DNA Damage Repair, Epigenetic Regulation, Metabolic Stress
Cytokine and Chemokine Signaling, Myeloid Compartment
Cell Proliferation, JAK-STAT Signaling, MAPK, Metabolic Stress, Notch Signaling, PI3K-Akt, TGF-beta Signaling, Wnt
Myeloid Compartment
Myeloid Compartment

Hypoxia, Metabolic Stress
Metabolic Stress
Costimulatory Signaling, Lymphoid Compartment, NF-kappaB Signaling
Antigen Presentation, Immune Cell Adhesion and Migration, Lymphoid Compartment

Cell Proliferation, DNA Damage Repair, Metabolic Stress, PI3K-Akt
TGF-beta Signaling
Hedgehog Signaling, Wnt Signaling
Antigen Presentation, Costimulatory Signaling, Immune Cell Adhesion and Migration, Interferon Signaling
Cell Proliferation
Cytokine and Chemokine Signaling, Myeloid Compartment
Cell Proliferation
Cell Proliferation, DNA Damage Repair
Cytokine and Chemokine Signaling, Hypoxia, JAK-STAT Signaling, Metabolic Stress
Hypoxia, Interferon Signaling, JAK-STAT Signaling
Autophagy, Costimulatory Signaling, Cytokine and Chemokine Signaling, Hypoxia, Immune Cell Adhesion and Migr

Antigen Presentation, Costimulatory Signaling, Immune Cell Adhesion and Migration, Interferon Signaling
Apoptosis, Autophagy, JAK-STAT Signaling, MAPK, PI3K-Akt
Epigenetic Regulation
Angiogenesis

Antigen Presentation, Hypoxia, Metabolic Stress

Myeloid Compartment
Costimulatory Signaling, Immune Cell Adhesion and Migration, Lymphoid Compartment, NF-kappaB Signaling
Hedgehog Signaling
Hypoxia, Metabolic Stress
Angiogenesis, Epigenetic Regulation, Metabolic Stress
NF-kappaB Signaling
MAPK, Metabolic Stress
Cytokine and Chemokine Signaling, Myeloid Compartment

Angiogenesis, Hypoxia, MAPK, PI3K-Akt
Angiogenesis, Matrix Remodeling and Metastasis, PI3K-Akt, TGF-beta Signaling

Cytokine and Chemokine Signaling, Lymphoid Compartment

Cytotoxicity
DNA Damage Repair
Myeloid Compartment
Angiogenesis
Apoptosis, Wnt Signaling
Antigen Presentation, Cytokine and Chemokine Signaling, Myeloid Compartment
Cell Proliferation, Epigenetic Regulation, JAK-STAT Signaling, Notch Signaling, PI3K-Akt, Wnt Signaling
Matrix Remodeling and Metastasis, PI3K-Akt
Hypoxia, Metabolic Stress
Angiogenesis, Cell Proliferation, Metabolic Stress, PI3K-Akt
Hypoxia, Metabolic Stress
Cell Proliferation, DNA Damage Repair
Cytotoxicity
NF-kappaB Signaling
Apoptosis
DNA Damage Repair
Costimulatory Signaling, Metabolic Stress
Cytotoxicity, JAK-STAT Signaling, Myeloid Compartment
Cell Proliferation
Cell Proliferation
Angiogenesis, Notch Signaling
Apoptosis, Cell Proliferation, DNA Damage Repair
Autophagy, Metabolic Stress
Hypoxia, Metabolic Stress
Cytotoxicity, Myeloid Compartment
Epigenetic Regulation, Metabolic Stress

Antigen Presentation, Cell Proliferation, Metabolic Stress
Apoptosis
Costimulatory Signaling, Interferon Signaling, Lymphoid Compartment
Cytotoxicity, Matrix Remodeling and Metastasis, Myeloid Compartment
Metabolic Stress
Costimulatory Signaling, Immune Cell Adhesion and Migration, Interferon Signaling, JAK-STAT Signaling, MAPK

probe.ID
NM_002416.2:2012
NM_005601.3:633
NM_001768.6:2029
NM_006144.3:215
NM_002985.2:277
NM_052941.4:1761
NM_000619.2:450
NM_001335.3:1075
NM_006419.2:209
NM_003897.3:204
NM_000878.2:1980
NM_000239.2:381
NM_001031680.2:3786
NM_007360.3:522
NM_018326.2:315
NM_002258.2:85
NM_000579.3:366
NM_001256370.1:1094
NM_006564.1:97
NM_002262.3:393
NR_024115.1:2175
NM_000732.4:127
NM_025239.3:235
NR_024168.1:2575
NM_002053.2:1838
NM_004324.3:330
NM_000491.3:816
NM_001558.2:150
NM_000206.1:595
NM_033423.3:705
NM_005816.4:1355
NM_001113755.2:645
NM_001565.3:122
NM_004221.4:358
NM_001767.3:683
NM_002209.2:3905
NM_001172129.1:325
NM_001037631.2:392
NM_001195797.1:135
NM_005409.4:333
NM_000639.2:605
NM_004079.3:685
NM_004972.2:455
NM_000397.3:1034
NM_002104.2:634
NM_001282426.1:1805

NM_000073.2:404
NM_002286.5:1736
NM_002460.1:325
NM_080921.3:99
NM_001561.5:567
NM_058216.2:15
NM_001278405.1:256
NM_000887.4:561
NM_000289.5:2672
NM_015991.3:636
NM_001775.2:280
NM_080921.3:258
NM_002982.3:303
NM_007315.3:239
NM_001171653.1:1879
NM_001244871.1:2475
NM_005356.4:1723
NM_001098175.1:1418
NM_001759.2:5825
NM_002003.4:969
NM_001223.4:1050
NM_001251.2:1140
NM_000442.4:2492
NM_001066.2:596
NM_172004.3:357
NM_002030.4:733
NM_002065.6:1485
NM_001278426.3:591
NM_004048.2:573
NM_012092.3:591
NM_000566.3:561
NM_002163.2:1245
NM_001184879.1:1015
NM_001207014.1:1850
NM_001243077.1:438
NM_001127628.1:488
NM_000660.4:1473
NM_000570.4:255
NM_001289023.2:864
NM_012483.3:540
NM_000211.3:1412
NM_005191.3:674
NM_152852.2:146
NM_000885.5:1019
NM_173799.2:1968
NM_000733.3:233
NM_002800.4:394

NM_005516.5:1287
NM_002121.5:987
NM_004513.4:1262
NM_001174105.1:1020
NM_005211.3:958
NM_016817.2:380
NM_018965.3:848
NM_000389.2:1975
NM_006137.6:440
NM_003733.2:1570
NM_000179.2:1015
NM_000594.3:1366
NM_004120.4:1744
NM_016388.3:578
ENST00000367367.1:131
NM_001242.4:326
NM_001243266.1:692
NM_001278318.1:486
NM_001015053.1:2169
NM_000593.5:1000
NM_002984.2:35
NM_001346456.1:1526
NM_005514.6:1247
NM_002117.4:898
NM_001078.3:2535
NM_001001392.1:566
NM_001256841.1:371
NM_001270508.1:3240
NM_000215.2:1715
NM_001276501.1:915
NM_001318787.1:484
NM_001243835.1:808
NM_024649.4:82
NM_005508.4:672
NM_001190945.1:712
NM_001350234.1:664
NM_005623.2:1012
NM_014308.3:3275
NM_001781.2:88
NM_032782.3:955
NM_001278182.1:1522
NM_021006.5:411
NM_000632.3:1400
NM_018833.2:912
NM_001778.3:413
NM_021642.3:60
NM_001079.3:1175

NM_002116.7:298
NM_001206924.1:1685
NM_001114937.2:418
NM_002341.1:330
NM_001061.4:709
NM_001145457.2:409
NM_000041.2:96
NM_003745.1:1022
NM_147162.1:400
NM_002124.3:1016
NM_004072.2:762
NM_000920.3:262
NM_001098479.1:285
NM_033554.2:857
NM_001033667.2:339
NM_001032409.1:805
NM_000958.2:975
NM_032206.4:3668
NM_197954.2:55
NM_203416.2:1784
NM_005533.4:729
NM_024070.3:1390
NM_002198.2:557
NM_001002273.1:870
NM_002185.3:1355
NM_001025159.2:1161
NM_005165.2:335
NM_005101.3:305
NM_006509.3:956
NM_001144925.2:1369
NM_006301.3:2680
NM_002118.3:20
NM_023914.2:2350
NM_004029.2:1705
NM_002199.3:1624
NM_001197122.1:607
NM_002543.3:254
NM_001005291.1:1392
NM_000633.2:1525
NM_006187.3:2354
NM_003263.3:696
NM_001253849.1:871
NM_007371.3:2621
NM_002037.5:2125
NM_014417.4:1310
NM_000616.4:1524
NM_181501.1:502

NM_000014.4:1685
NM_001244.3:399
NM_023068.3:4559
NM_001123041.2:438
NM_002405.3:1768
NM_001134836.1:600
NM_001001523.1:2839
NM_002308.3:1206
NM_001699.4:1535
NM_016562.3:4120
NM_002128.5:3400
NM_004958.3:3444
NM_001163034.1:3139
NM_001145645.2:142
NM_002438.2:525
NM_001171171.1:2625
NM_018295.3:377
NM_002468.3:2145
NM_021975.3:1990
NM_001034116.1:1258
NM_003824.3:810
NM_003177.5:808
NM_000576.2:1122
NM_002200.3:1845
XM_011542270.1:326
NM_000077.4:1052
NM_030641.3:289
NM_002995.2:266
NM_001145306.1:1373
NM_005903.5:5007
NM_004994.2:2011
NM_002737.2:818
NM_080657.4:1057
NM_024756.2:3040
NM_001547.4:1995
NM_020529.2:1361
NM_000757.5:251
NM_173091.2:1840
NM_001130823.2:2500
NM_003188.3:1178
NM_001146055.1:480
NM_022168.3:3250
NM_014905.4:165
NM_003392.3:987
NM_021950.3:161
NM_004159.4:1080
NM_022162.1:4080

NM_000760.3:2066
NM_002029.3:625
NM_006288.3:420
NM_000251.1:2514
NM_014862.3:1133
NM_002162.3:1225
NM_001127702.1:963
NM_001100122.1:1686
NM_005532.3:390
NM_001330347.1:2169
NM_005027.3:344
NM_019111.3:335
NM_001136017.2:760
NM_002080.3:674
NM_001146103.1:1188
NM_017654.3:490
NM_001272042.1:2451
NM_022153.1:185
NM_001114614.1:328
NM_017734.4:224
NM_006120.3:396
NM_004107.4:1260
NM_000600.3:484
NM_004418.3:1235
NM_003377.3:687
NM_001031683.3:712
NM_000245.2:1488
NM_005627.3:1621
NM_001783.3:618
NM_015259.4:1190
NM_001145847.1:596
NM_001270949.1:1940
NM_022750.3:1843
NM_000577.3:480
NM_000321.2:2769
NM_001130725.1:426
NM_001770.4:1770
NM_002164.5:52
NM_004322.3:963
NM_006252.3:4014
NM_003809.2:339
NM_000917.3:805
NM_017617.3:2999
NM_003190.4:1082
NM_000585.4:715
NM_003200.3:276
NM_002855.4:357

NM_001289999.1:1466
NM_001111045.1:892
NM_001024736.1:2119
NM_013252.2:615
NM_000247.1:550
NM_182948.2:805
NM_002988.3:45
NM_002801.3:249
NM_003051.3:635
NM_002038.3:410
NM_005534.3:1963
NM_004448.2:2405
NM_015441.1:2920
NM_004936.3:906
NM_002079.2:568
NM_015177.1:4820
NM_001270930.1:1675
NM_004985.3:1790
NM_004419.3:675
NM_001034.1:490
NM_025216.2:1781
NM_001171623.1:2212
NM_138287.3:1543
NM_001135768.2:448
NM_001174064.1:1756
NM_000535.6:894
NM_024408.3:2842
NM_001284403.1:326
NM_001008541.1:2037
NM_001199723.1:802
NM_001135652.2:516
NM_001206796.2:1533
NM_002964.4:365
NM_006084.4:723
NM_138441.2:1198
NM_001077494.3:1986
NM_006015.4:1670
NM_000267.3:1260
NM_003862.2:1030
NM_001144999.2:2701
NM_000972.2:657
NM_001142593.2:958
NM_181054.2:930
NM_000063.5:730
NM_005764.3:229
NM_000582.2:760
NM_018131.3:570

NM_001330636.1:865
NM_002485.4:1046
NM_002354.2:499
NM_003641.3:536
NM_000366.5:830
NM_000546.2:1330
NM_006871.3:1072
NM_000629.2:461
NM_003884.4:1180
NM_000093.4:1133
NM_001206567.1:2351
NM_003486.6:2011
NM_003141.3:1190
NM_001243211.1:392
NM_000495.4:1714
NM_003335.2:580
NM_001255.1:915
NM_002214.2:2609
NM_004556.2:876
NM_001098209.1:1938
NM_005985.3:960
NM_001130916.2:3011
NM_001322977.1:191
NM_001797.2:152
NM_001316306.1:1982
NM_001144822.1:42
NM_000051.3:393
NM_015897.2:454
NM_000314.6:6696
NM_006218.2:3296
NM_003820.3:503
NM_001001548.2:705
NM_005228.3:2760
NM_005931.3:1387
NM_001165136.1:883
NM_004052.3:116
NM_002610.3:401
NM_000615.6:1134
NM_002119.3:3075
NR_048564.1:949
NM_005419.2:1965
NM_001284244.1:241
NM_182943.2:2588
NM_001278.4:3263
NM_001164319.1:8980
NM_002996.3:1850
NM_001024847.2:870

NM_001674.3:1487
NM_001135599.3:1972
NM_001008540.1:1060
NM_000578.3:1679
NM_001244134.1:493
NM_001099856.4:1719
NM_004417.3:1428
NM_003842.3:565
NM_001285439.1:117
NM_001193520.1:3097
NM_014805.3:1322
NM_152275.3:2492
NM_007294.3:4677
NM_005406.2:3796
NM_001196.3:1604
NM_006435.2:390
NM_002208.4:3647
NM_001297562.1:332
NM_014791.2:365
NM_138636.4:2210
NM_001330491.1:2559
NM_002524.4:2168
NM_003294.3:556
NM_182919.3:2552
NM_181050.1:2742
NM_001126336.2:394
NM_002417.2:2005
NM_002033.3:1151
NM_000876.2:7820
NM_000107.2:1556
NM_001018112.1:580
NM_005271.2:2680
NM_006037.3:3742
NM_001243076.2:2707
NM_024608.2:1675
NM_003821.5:1306
NM_001935.3:1861
NM_001323611.1:1085
NM_005242.4:1388
NM_001065.2:515
NM_003810.3:155
NM_001042724.1:1120
NM_000565.3:846
NM_032043.2:1570
NM_001146282.1:951
NM_000125.2:1595
NM_001018073.2:1307

NM_001172699.1:1010
NM_001228.4:762
NM_001792.3:3067
NM_005732.3:1357
NM_198282.1:725
NM_007108.2:800
NM_001291880.1:1169
NM_001289067.1:191
NM_003883.3:1464
NM_001318204.1:674
NM_000059.3:1373
NM_002412.3:323
NM_014584.2:963
NM_000627.3:1970
NM_031866.1:890
NM_001044305.2:823
NM_002822.4:1574
NM_001448.2:820
NM_002227.1:285
NM_004095.3:673
NM_001190720.2:1891
NM_001039667.2:1088
NM_005163.2:2207
NM_000201.2:2253
NM_032427.1:4125
NM_002423.3:265
NM_001145646.1:541
NM_025079.2:1379
NM_000584.3:170
NM_006027.3:820
NM_000365.5:153
NM_001145144.1:648
NM_024908.3:1875
NM_001017973.1:1748
NM_003998.3:2920
NM_005343.3:842
NM_133487.3:1570
NM_006437.3:1399
NM_000043.3:90
NM_005524.2:860
NM_032991.2:1002
NM_000034.3:2049
NM_000228.2:3350
NM_000076.2:1605
NM_006516.2:264
NM_004360.3:379
NM_014373.1:760

NM_004369.3:3293
NM_006845.2:1020
NM_005194.3:1731
NM_001168.2:1215
NM_001128128.2:2292
NM_018685.2:240
NM_002105.2:521
NM_002090.2:467
NM_002467.3:1615
NM_001242589.2:101
NM_001657.3:717
NM_000978.3:100
NM_004566.3:1925
NM_001174097.2:1200
NM_148970.1:151
NM_002120.3:124
NM_001124.2:1413
NM_001290230.1:1543
NM_001546.3:2048
XM_011545241.2:1015
NM_002122.3:258
NM_031966.2:710
NM_002089.1:435
NM_016343.3:9260
NM_000249.2:1605
NM_003150.3:2936
NM_000416.2:1025
NM_001242466.1:889
NM_003268.5:2570
NM_002123.3:384
NM_001191.3:1421
NM_001330384.1:2083
NM_005860.2:255
NM_002526.3:3526
NM_198156.1:420
NM_001172.3:512
NM_002965.3:137
NM_001250.5:166
NM_002048.2:948
NM_001135239.1:1053
NM_004456.4:1596
NM_002546.3:572
NM_005923.3:3371
NM_001130046.1:295
NM_018009.4:1425
NM_002019.4:530
NM_003246.2:3816

NM_001100812.1:743
NM_001142930.1:1552
NM_001031709.2:726
NM_001130852.1:657
NM_003747.2:1948
NM_006770.3:1435
NM_001300927.1:6607
NM_001127510.2:462
NM_001511.3:743
NM_053056.2:857
NM_005562.2:1027
NM_000188.2:3355
NM_001238.1:1155
NM_000189.4:2883
NM_001256849.1:1356
NM_030964.3:1900
NM_003808.3:810
NM_003265.2:37
NM_014176.1:50
NM_015366.3:1635
NM_002309.4:3740
NM_001790.4:1056
NM_001071.1:395
NM_000214.2:3509
NM_000057.3:1434
NM_001283012.1:1141
NM_001428.3:782
NM_001040023.1:3355
NM_145902.2:906
NM_005504.6:1060
NM_007019.2:445
NM_003804.3:1304
NM_001964.2:2034
NM_001777.3:901
NM_001320635.1:753
NM_001330437.1:228
