# Supplementary material for: Long-Term Follow-Up of Gemogenovatucel-T (Vigil) Survival and Molecular Signals of Immune Response in Recurrent Ovarian Cancer
Source: Vaccines (Basel). 2021 Aug 12;9(8):894. doi: 10.3390/vaccines9080894 (PMC8402348; doi:10.3390/vaccines9080894)
Supplement: Supplementary file 1 [file vaccines-09-00894-s001.zip › vaccines-1279453 after proof supl.pdf]

Supplemental File 1 Table S1. Immune Pathway Results

Sample	FANG-OV-1049	FANG-OV-1086	FANG-OV-1107	FANG-OV-1122
TIS status	TIS-high	TIS-high	TIS-high	TIS-high
ELISPOT status	ELISPOT-positive	ELISPOT-positive	ELISPOT-positive	ELISPOT-positive
Response status	GR	GR	GR	PR
TIS	8.062	8.021	7.279	8.489
APM	5.824	6.156	5.465	6.042
Apoptosis	5.366	4.701	5.011	4.447
ARG1	3.438	0.3919	3.793	1.097
B Cells	7.677	4.058	6.887	6.016
B7-H3	3.939	5.603	4.728	4.311
CD45	6.156	5.976	6.249	5.58
CD8 T Cells	6.224	5.395	5.931	5.47
CTLA4	9.135	7.474	9.179	8.761
Cytotoxic Cells	6.945	7.328	7.361	7.004
Cytotoxicity	6.738	6.975	7.387	7.232
DC	5.59	4.156	4.326	3.278
Endothelial Cells	4.302	2.546	3.563	0.9151
Exhausted CD8	7.106	6.8	7.151	6.557
Glycolytic Activity	5.466	4.903	5.652	4.994
Hypoxia	5.359	4.113	6.515	4.721
IDO1	6.443	5.713	3.238	7.331
IFN Downstream	5.859	7.087	5.272	6.838
IFN Gamma	7.81	8.239	6.497	8.635
IL10	8.358	3.547	7.364	5.331
Immunoproteasome	6.132	5.85	4.454	5.85
Inflammatory Chemokines	5.933	6.438	6.341	6.876
Lymphoid	6.841	6.717	6.745	6.517
Macrophages	5.619	6.48	5.583	5.566
MAGEs	1.615	-0.9018	1.255	-1.045
Mast Cells	3.374	0.6088	3.751	1.448
MHC2	7.248	6.49	6.13	5.959
Myeloid	6.043	5.908	5.833	5.516
Myeloid Inflammation	4.41	2.765	4.266	2.969
Neutrophils	5.946	4.601	5.316	4.134
NK CD56dim	6.007	4.428	6.49	4.453
NK Cells	7.458	6.796	6.702	5.767
NOS2	3.818	0.09464	0.9109	0.4376
PD-1	6.75	5.2	5.95	5.534
PD-L1	4.979	4.809	4.949	4.553
PD-L2	6.204	5.755	6.088	5.618
Proliferation	6.439	4.401	6.051	7.211
Stroma	2.979	1.848	3.203	1.294
T Cells	6.624	6.763	7.04	6.093
TGF-Beta	6.348	4.806	4.039	3.56
TH1 Cells	6.975	5.667	6.706	4.077
TIGIT	6.896	6.026	7.614	7.094
Treg	5.101	4.963	7.653	5.913

FANG-116<br>TIS-high<br>ELISPOT-positive<br>GR	FANG-OV-1021<br>TIS-high<br>ELISPOT-positive<br>GR	FANG-OV-1024<br>TIS-high<br>ELISPOT-negative<br>GR	FANG-OV-1025<br>TIS-high<br>ELISPOT-positive<br>PR	FANG-OV-1091<br>TIS-low<br>ELISPOT-positive<br>GR
9.329	7.86	6.309	9.421	5.508
6.7	6.404	4.387	6.88	4.71
4.292	5.437	3.885	4.752	3.732
1.183	-0.2895	-1.557	1.571	-0.1266
6.816	5.078	3.844	8.504	3.974
4.301	4.442	3.357	4.152	5.354
6.719	5.28	4.083	7.612	3.955
6.142	5.655	3.732	6.402	2.905
9.103	9.003	6.463	9.906	5.156
7.367	7.056	5.737	8.436	3.991
7.25	6.892	5.755	8.161	4.14
4.712	2.797	3.356	4.072	2.684
1.282	1.411	1.531	2.61	1.932
7.266	6.288	5.142	8.332	4.266
5.77	5.579	5.123	4.663	5.134
5.431	5.13	5.111	3.981	5.124
7.553	7.47	2.829	7.342	3.696
7.267	5.666	5.701	7.914	5.199
9.522	7.648	6.58	9.725	5.227
5.56	5.188	5.406	8.048	4.836
6.823	6.611	3.858	6.558	3.864
7.31	5.339	5.892	8.274	5.127
7.062	6.331	4.844	7.904	4.131
6.708	4.98	5.118	7.577	5.015
-0.3153	-1.651	-1.892	-0.408	-1.081
0.3946	1.287	-0.1439	1.063	1.111
6.962	7.155	4.418	7.33	6.119
6.099	5.543	4.413	6.594	4.904
3.958	5.781	2.629	4.444	3.422
5.066	4.124	3.345	4.883	3.645
4.268	3.748	3.502	5.613	3.315
6.826	6.293	5.655	8.366	4.996
-0.1147	-1.587	-0.2689	1.689	0.161
5.232	4.551	3.699	6.444	2.129
5.367	3.306	2.955	5.243	2.307
6.527	4.941	4.635	6.801	3.75
6.601	5.926	6.653	4.709	5.354
1.7	2.576	1.71	2.326	2.299
6.972	5.91	4.236	7.858	3.611
4.577	3.971	2.7	5.702	2.672
5.195	3.4	3.455	5.261	3.148
8.452	7.579	4.929	8.29	3.555
6.831	6.826	3.014	7.364	4.029

FANG-032	FANG-037	FANG-046
TIS-low	TIS-low	TIS-low
ELISPOT-negative	n/a	n/a
PR	PR	PR
4.438	5.475	5.794
2.961	5.109	2.949
4.19	4.337	4.119
-1.816	0.8422	0.02183
3.585	4.778	6.321
5.871	4.485	4.916
2.343	3.628	4.414
1.613	2.3	3.465
4.104	4.692	7.222
3.129	4.492	4.728
2.973	4.151	4.442
2.163	1.991	1.995
2.774	2.275	1.831
4.11	4.573	4.786
5.367	6.09	5.266
5.275	6.163	4.764
0.5656	3.911	3.673
4.251	6.27	5.217
3.214	5.769	4.827
5.04	4.413	2.592
3.017	5.927	4.792
3.193	4.713	4.305
3.31	4.369	5.195
3.154	3.744	3.367
-0.7829	-1.473	-2.132
0.2674	0.1371	0.1584
4.314	4.472	4.246
3.221	4.463	3.24
1.728	4.325	1.802
2.8	3.234	2.356
2.679	3.034	3.256
2.551	5.737	4.884
0.4716	-0.0401	-0.8604
3.118	2.776	3.956
1.954	3.036	3.202
2.764	3.717	3.204
6.084	5.197	7.012
2.148	1.871	1.145
2.757	3.833	5.345
1.593	2.344	3.202
4.196	3.532	3.934
2.135	3.895	6.605
3.492	4.243	4.745
